# Supplementary material for: Tyrosine 121 moves revealing a ligandable pocket that couples catalysis to ATP-binding in serine racemase
Source: Commun Biol. 2022 Apr 11;5:346. doi: 10.1038/s42003-022-03264-5 (PMC9001717; doi:10.1038/s42003-022-03264-5)
Supplement: Supplementary file 2 — Supplementary Information [file 42003_2022_3264_MOESM2_ESM.pdf]

SUPPLEMENTARY MATERIAL FOR:

Tyrosine 121 moves revealing a ligandable pocket that couples catalysis to ATP-binding in serine racemase.

Chloe R. Koulouris[a], Sian E. Gardiner[b], Tessa K. Harris[b], Karen T. Elvers[b], S. Mark Roe[c], Jason Gillespie[b], Simon Ward[b], Olivera Grubisha [b], Robert A. Nicholls[d], John R. Atack[b], and Benjamin D. Bax [b‡]

* To whom correspondence should be addressed (email: AtackJ@cardiff.ac.uk or baxb@cardiff.ac.uk)

Supporting information content:

Supplementary Table 1. Data collection and refinement statistics for seven human SR crystal structures.
Supplementary Table 2. List of serine racemase (SR) and serine dehydratase (SDH) structures from the PDB.
Supplementary Table 3. Twelve XChem compounds with electron density in at least one Tyr 121 pocket.
Supplementary Table 4. IC50s for compounds
Supplementary Table 5. Phi-psi angles for the β3-α4 loop and for the β6 strand regions**.**
Supplementary Table 6. Summary of SR inhibitors and physiological modulators

Supplementary Figure 1. Chemical structures of PLP, PLP-Lys56 and PLP-L-Ser, PLP-D-Ser and PLP-planar intermediate
Supplementary Figure 2. Comparison of 'open' and 'closed' structures of human and yeast serine racemase
Supplementary Figure 3. Serine racemase chemistry and lysino-d-alanyl and gem-diamine structures.
Supplementary Figure 4. Malonate binding sites in human SR crystal structures (6ZSP and 3L6B)
Supplementary Figure 5. Tyr121 can form hydrogen bonds to ATP or main-chain carbonyl of Ser84
Supplementary Figure 6. The side-chain of Cys 113 is accessible in the 'open' human SR structure but is buried in the 'closed' structure
Supplementary Figure 7. Serine racemase sequence alignment
Supplementary Figure 8. Superpositions of human *holo* and ATP/malonate-bound SR with yeast orthologues
Supplementary Figure 9. Superpositions of closed SR ATP/malonate-bound structures with open SR subunits A and C
Supplementary Figure 10. The hydrophobic core of the dimer interface in open and closed hSR structures
Supplementary Figure 11. ATP dependent activity data on Wild-type, Y121A, Y121F and Y121G mutants
Supplementary Figure 12. Inhibition data on Wild-type, Y121A, Y121F and Y121G mutants of hSR.
Supplementary Figure 13. Roles of lysine 56 and serine 84 in proton exchange in human serine racemase
Supplementary Figure 14. Does Arginine 135 protonate Ser 84 via the side-chain of the substrate serine?
Supplementary Figure 15. Serine dehydratase and serine racemase sequence alignment.
Supplementary Figure 16. OMIT maps of ATP, malonate, and bound XChem ligands
Supplementary Figure 17. OMIT maps of XChem ligands bound to SR

Supplementary Discussion. The evolution and regulation of the activity of human serine racemase in the CNS
Supplementary References
SEE ALSO Three supplementary movies (Additional Supplementary Files)

Supplementary Table 1. Data collection and refinement statistics for seven human SR crystal structures (including five XCHem soaks).

Parameter/ ligand sites	Active site/ ATP	No ligand XChem control	Small domain Y121	Sd. Y121 + L. dom. D238	Small domain T81	Dimer interface W278	Dimer interface L25
PDB ID (resolution) LIGAND/cmpnd no.	6ZSP (1.60 Å) Mal/ATP	6ZUJ (1.80 Å)	7NBG (1.53 Å) 1-x0495	7NBH (1.77 Å) 2-x0406	7NBC (1.71 Å) 13-x0430	7NBD (1.86 Å) 14-x0478	7NBF (1.60 Å) 15-x0360
Space group	$P2_12_12_1$	$P2_1$	$P2_1$	$P2_1$	$P2_1$	$P2_1$	$P2_1$
a (Å), b (Å), c (Å)	53.94, 81.19, 134.46	48.06, 155.00, 85.65	48.11, 154.76, 85.40	48.18 154.88 85.48	48.15 154.86 85.48	48.01 154.85 85.45	48.11 154.94 85.54
α=90°, β, γ=90°	90°	98.3°	98.14°	98.04°	98.08°	98.04°	97.96°
Data collection parameters							
Wavelength	0.978	0.91587	0.91587	0.91587	0.91587	0.91587	0.91587
Resolution range high res. shell.	50.1–1.60 1.63–1.60	84.7–1.80 1.83–1.80	77.4–1.53 1.57–1.53	51.6–1.77 1.82–1.77	77.4–1.71 1.74–1.71	84.6–1.86 1.97–1.86	84.7–1.60 1.63–1.60
Total no. of reflections	304094 (7908)	381617 (19294)	575680 (20850)	402122 (18838)	440513 (18933)	333133 (42883)	517542 (20057)
No. of unique reflections	76688 (3151)	111815 (5444)	177718 (11899)	116780 (8546)	130785 (6420)	96340 (12195)	158956 (7536)
Completeness (%)	97.5 (81.0)	97.8 (95.61)	95.9 (87.0)	97.1 (96.3)	98.2 (97.2)	94.0 (82.0)	97.6 (93.4)
Multiplicity	4.0 (2.5)	3.4 (3.5)	3.2 (2.6)	3.4 (3.3)	3.4 (2.9)	3.5 (3.5)	3.3 (2.7)
I/σI	9.2 (1.3)	9.5 (1.7)	9.0 (1.0)	8.2 (1.0)	8.3 (1.0)	8.9 (1.1)	8.9 (1.1)
R_{merge} (I)	0.069 (0.577)	0.068 (0.703)	0.052 (0.804)	0.072 (1.048)	0.067 (0.948)	0.073 (0.912)	0.059 (0.658)
CC1/2	0.999 (0.512)	0.9973 (0.651)	0.998 (0.453)	0.997 (0.302)	0.997 (0.305)	0.998 (0.470)	0.997 (0.383)
R-meas	0.079 (0.705)	0.081 (0.829)	0.075 (1.002)	0.098 (1.424)	0.093 (1.306)	0.100 (1.235)	0.082 (0.889)
Refinement res. range (Å)	20–1.6	84.1–1.8	77.4–1.53	47.7–1.77	77.6–1.71	84.6–1.86	84.7–1.60
No. of refls, working (test)	72966 (3722)	106173 (5512)	168961 (8706)	110878 (5859)	124213 (6527)	91467 (4830)	150883 (7962)
Final Rcryst	0.192	0.179	0.183	0.172	0.174	0.176	0.172
Final Rfree	0.227	0.220	0.201	0.202	0.201	0.207	0.198
No. of non-H atoms							
Protein	5267	9879	10369	10201	10079	10155	10106
Ions	4	11	15	16	12	10	14
Ligand/other	125	202	239	294	184	245	284
Water	333	974	946	989	867	1069	929
Total	5729	11066	11570	11500	11142	11479	10503
R.M.S. deviations							
Bonds (Å)	0.006	0.006	0.004	0.009	0.007	0.010	0.013
Angles (°)	1.348	1.319	1.151	1.446	1.356	1.669	1.882
Average B factor (Å^2)	21.9	34.2	28.6	35.2	32.4	37.9	29.0
Protein	21.7	34.4	26.7	34.4	32.2	37.3	28.7
Ion	15.1	36.4	40.3	47.8	39.3	41.2	31.6
Ligand	22.7	38.7	34.8	44.6	35.5	39.9	28.4
Water	32.3	42.1	39.4	45.8	41.5	49.7	38.6
Ramachandran plot							
Favoured regions (%)	98.2	97.8	98.0	98.1	96.3	97.4	97.7
Additionally allowed (%)	1.8	1.1	1.8	1.8	3.5	2.5	2.3
Outliers (%)	0.0	1.1	0.2	0.1	0.2	0.1	0.0

Supplementary Table 2. List of serine racemase (SR) and serine dehydratase (SDH) structures from the PDB

PDB code (resolution)	Species	Protein	Ligands	Metal ion site metal	Reference
**6ZSP** (1.60 Å)	*H. sapiens*	SR	PLP, MAL, ATP	Mg^{2+}	Current study
**6ZUJ** (1.80 Å)			PLP	Ca^{2+}	Current study
**7NBC** (1.71 Å)			PLP, x0430	Ca^{2+}	Current study
**7NBD** (1.86 Å)			PLP, x0478	Ca^{2+}	Current study
**7NBF** (1.60 Å)			PLP, x0306	Ca^{2+}	Current study
**7NBG** (1.53 Å)			PLP, x0495	Ca^{2+}	Current study
**7NBH** (1.77 Å)			PLP, x0406	Ca^{2+}	Current study
**6SLH** (1.89 Å)			PLP	Mg^{2+}	[1]
**5X2L** (1.81 Å)			PLP	Mg^{2+}	[2]
**3L6B** (1.50 Å)			PLP, MAL	Mn^{2+}	[3]
**3L6C** (2.20 Å)	*R. norvegicus*	SR	PLP, MAL	Mn^{2+}	[3]
**3HMK** (2.10 Å)			PLP	Mn^{2+}	[3]
**1WTC** (1.90 Å)	*S. pombe*	SR	PLP, AMPPCP	Mg^{2+}	[4]
**1V71** (1.70 Å)			PLP	Mg^{2+}	[4]
**2ZR8** (2.20 Å)			PDD, L-SER	Mg^{2+}	[4]
**2ZPU** (1.70 Å)			PDD	Mg^{2+}	[5]
**5CVC** (2.09 Å)	*Z. mays*	SR	PLP	Mg^{2+}	[6]
**1PWE** (2.80 Å)	*R norvegicus*	SDH	-	-	[7]
**1PWH** (2.60 Å)			PLV	K^+	[7]
**1P5J** (2.50 Å)	*H. sapiens*	SDH	PLP	H_2O	[8]
**4H27** (1.30 Å)			PLP	H_2O	[9]
**2RKB** (2.80 Å)		SDH like-1	PLP	K^+	[10]

AMPPCP, Adenosine 5'-[β,γ-methylene]triphosphate (non-hydrolysable analogue of ATP); ATP, Adenosine triphosphate; L-SER, L-Serine; MAL, malonate; PDD, N-(5'-phosphopyridoxyl)-D-alanine; PLP, pyridoxal 5'-phosphate; PLV, PLP-O-methylserine aldimine

Supplementary Table 3. Twelve XChem compounds with electron density in at least one Tyr 121 pocket.

PDB code<br>Cmp no.<br>(Resol.)	SMILES	Picture	Initial rank*/ Comments
7NBG<br>**1**-x0495<br>(1.53Å)	CC(=O)NCCC=1C=CC=CC1C	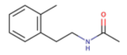	A. Methylbenzene in pocket in subunits A and B (and half occupancy in D. Tyr 121 in pocket in C.
7NBH<br>**2**-x0406<br>(1.77Å)	O=C(NCC1=NC=2C=CC=CC2N1)<br>C3=CC=CO3	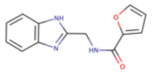	B. Benzimidazole in subunits A,B,D. Tyr 121 in pocket in subunit C. Second site.
**3**-x0458<br>(1.85Å)	NC=1C=CC=CC1N2CCOCC2	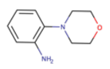	B. Aniline in pocket in A,B subunits. Aniline nitrogen H-bonds to MC, C=0 of Ser 84 and Gly 85.?
**4**-x0482<br>(1.71Å)	CC(=O)NC1CNC=2C=CC=CC2C1	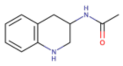	B. Benzene in pocket in subunits A and B, nitrogen of tetrahydroquinoline may make H-bond to Ser 84 C=O.
**5**-x0501<br>(1.82Å)	NC(=O)C=1C=NC(=NC1)C(F)(F)F	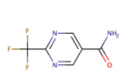	D. Tyrosine 121 in pocket in subunits A and B, electron density in subunits C and D suggests half occupancy compound with carboxamide in pocket mimicking hydroxyl of Tyr 121?
**6**-x0513<br>(1.45Å)	CNCC1COC=2C=CC=CC2O1	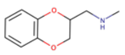	A. Benzene in pocket in subunits A,B,D. Tyr 121 in pocket in subunit C.
**7**-x0109<br>(1.73Å)	O=C(CCCC=1C=CC=CC1)N2CCO<br>CC2	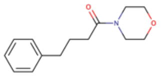	C. Benzene in pocket in subunits A,B,D. Tyr 121 in pocket in subunit C.
**8**-x0127<br>(1.87Å)	CCCC1=NN=C(NC(=O)C=2C=CC=<br>CN2)S1	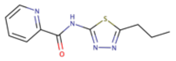	D. Pyridine in pocket in subunit D.
**9**-x0154<br>(1.83Å).	CN1N=CC=C1C(=O)NCC2=CC=C<br>S2	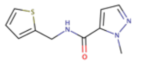	D. Density in pocket for one end of cmpnd in subunit B and D.
**10**-x0302<br>(2.23Å)	CC(CS(=O)(=O)N)C=1C=CC=CC1	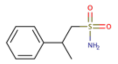	D. Benzene in pocket in subunit A.
**11**-x0431<br>(1.51Å)	CS(=O)(=O)CC(O)C=1C=CC=CC1	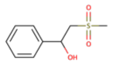	B. Benzene in pocket in subunits A and B.
**12**-x0437<br>(1.74Å)	NCC1=CC(=NO1)C=2C=CC=CC2	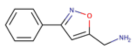	D. Benzene in pocket in subunits A,B,D. Tyr 121 in pocket in subunit C.

* Initial rank from examination of XChem electron density/fit; A = best, D = worst.

Supplementary Table 4. IC50s for compounds. Human SR inhibition data (see methods for details) is shown for some of the fifteen fragments from the XChem screen. ND = No data. Note - compounds 1-12 bind in Y121 pocket. Note only 3 compounds, 3-x0458, 4-x0482 and 13-x0430 showed 100% inhibition at maximum concentration used in assay (see supplementary fig 12 for raw data). Other compounds have estimated IC50s calculated by extrapolating from observed inhibition (see supplementary fig. 12 - for curves).

Cmpn no	Name or DSiP number	Structure	IC50 (mM)			
			WT	Y121A	Y121F	Y121G
1-x0495	Z52314092	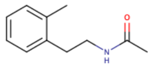	~19.5 ± 4.7	~23.6 ± 6.2	~18.7 ± 0.8	ND
2-x0406	Z26781964	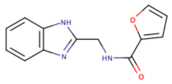	~3.7 ± 0.6	~8.4 ± 0.4	~7.8 ± 0.4	~7.4 ± 0.7
3-x0458	Z56785490	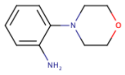	0.0106 ± 0.0014	0.0094 ± 0.0031	0.0123 ± 0.0005	0.0234 ± 0.0008
4-x0482	Z1492796719	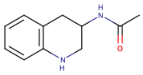	0.0051 ± 0.0008	0.0033 ± 0.0004	0.0032 ± 0.0001	0.0044 ± 0.0022
5-x0501	Z1745658474	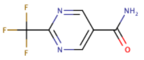	ND	ND	ND	ND
6-x0513	Z1891773476	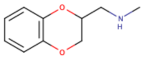	ND	ND	ND	ND
7-x0109	Z419995480	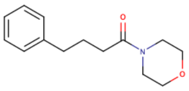	~12.5 ± 2	~22.3 ± 1.6	~20.7 ± 1.8	~26.0 ± 3.3
8-x0127	Z52425517	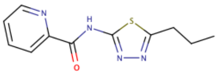	ND	ND	ND	ND
9-x0154	Z915492990	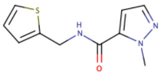	ND	ND	ND	ND
10-x0302	Z1407673036	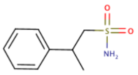	ND	ND	ND	ND
11-x0431	Z822382694	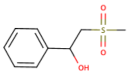	ND	ND	ND	ND
12-x0437	Z425449682	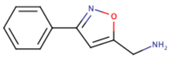	~25.3 ± 2.5	~37.6 ± 11.2	~29.9 ± 0.6	~26.25 ± 3.8
13-x0430	Z2856434779	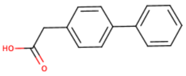	0.9 ± 0.3	2.15 ± 0.3	1.03 ± 0.6	ND
14-x0478	Z235449082	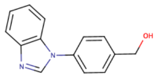	~6.9 ± 0	~7.85 ± 0.8	~7.4 ± 1.1	ND
15-x0306	Z126932614	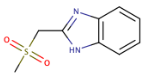	~12.8 ± 2.4	~16.05 ± 0.4	~18.7 ± 0.8	ND

Supplementary Table 5. Phi-psi angles for the β3-α4 loop and for the β6 strand regions**.**

	Holoenzyme (6ZUJ).				Malonate/ATP (6ZSP)			
	A-subunit		C-subunit		A-subunit		B-subunit	
	phi	psi	phi	psi	phi	psi	phi	psi
THR 81	-153	157	-138	148	-158	178	-157	176
**HIS 82**	-92	-9	-89	-27	-117	150	-117	148
SER 83	-97	126	-78	131	-162	146	-163	145
SER 84	-107	0	-102	3	-76	-11	-75	-10
**GLY 85**	-97	-128	-92	-153	150	-45	150	-44
ASN 86	-57	-34	-60	-41	-71	-47	-70	-49
HIS 87	-71	-45	-61	-50	-61	-38	-62	-37
GLY 148	-94	176	-97	-178	-84	-175	-85	-175
ILE 149	-109	136	-123	156	-113	123	-112	129
MET 150	-88	129	-116	148	-81	130	-82	127
VAL 151	-121	103	-132	117	-124	98	-119	96

Supplementary Table 6. Summary of SR inhibitors and physiological modulators

Group	Compound	Inhibition mode	Reference
**Serine analogues**	L-serine-*O*-sulfate	Competitive/ substrate mimetic	11-13
	L-cysteine-*S*-sulfate		11
	β-chloro-L-alanine		11,13
	Homocysteic acid		11,13
**Aspartate analogues**	L-aspartate	Competitive	11
	L-asparagine		11
	L-erythro-3-hydroxyaspartate		11
	L-aspartate β-hydroxamate		14
**Carboxylic acids**	Malonic	Competitive	11
	Succinic		11
	Maleic		11
	L-malic		11
	Tartaric		11
	Dihydroxyfumaric		11
	Oxaloacetic acid	Product inhibition	11,13
**Carboxylate-containing compounds**	Various; see reference	Competitive	15
**Malonate derivatives**	2-aminomalonate	Competitive	16
	2-fluoromalonate		16
	2-hydroxymalonate		16,17
	2,2-dichloromalonate		17
	2,2-bis(hydroxymethyl)malonate		17
**Substrate-product analogues**	α-(hydroxymethyl)serine	Substrate-product inhibition	18
**Cyclopropane carboxylic acid derivatives**	1,2-cyclopropanedicarboxylic acid	Competitive; related to malonate	19
**Peptidomimetic inhibitors**	Various; see references	Malonate site/ dimer interface	2,20-22
**Plant derivatives**	Madecassoside	Competitive	23
**Non-specific inhibitors**	Sulfhydryl compounds (e.g. L-cysteine)	PLP modification	13
	Hydroxamic acids		14
**Physiological modulators**	PIP2	Inhibition (noncompetitive); Interferes with ATP binding	24
	GRIP	Activation; binds to SR	25
	PICK1	Inhibition; binds to SR and induces its PPN by PKC	26
	Golga3	Increases SR levels by preventing ubiquitylation	27
	Nitric oxide	Inhibition; induces S-nitrosylation of SR	28
	NADH[9] + analogues	Inhibition; promotes ATP dissociation	29,30
	Stargazin/PSD-95	Inhibition; facilitates cell membrane localization of SR	31
	DISC1	Stabilises SR and prevents its degradation	32

ATP, adenosine triphosphate; DISC1, Disrupted-In-Schizophrenia-1; Golga3, Golgin subfamily A member 3; GRIP, glutamate receptor-interacting protein; NADH, nicotinamide adenine dinucleotide; PICK1, protein interacting with C-kinase; PIP2, phosphatidylinositol 4,5-bisphosphate; PSD-95, postsynaptic density 95 kDa

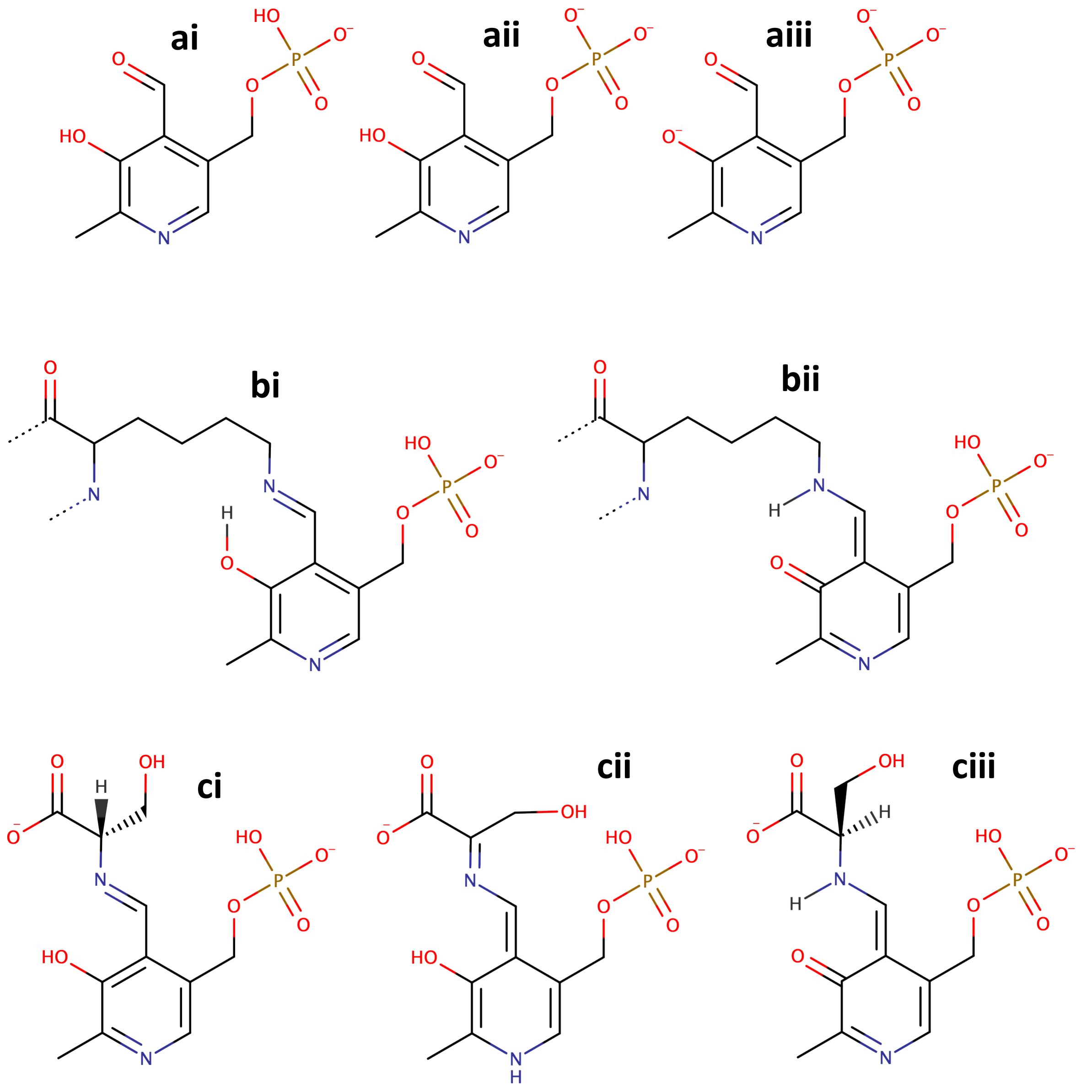

Supplementary Figure 1. **Chemical structures of PLP, PLP-Lys56 and PLP-L-Ser, PLP-D-Ser and PLP-planar intermediate.** (**ai, aii, aiii**) Chemical structures of three different protonated forms of pyridoxal -5' phosphate (PLP). The minus-1 form is predicted to be most stable below pH 6.8 (**ai**), the minus-2 form between pH 6.8–8.0 (**aii**), and the minus-3 form above pH 8.0 (**aiii**). (**bi, bii**) Two tautomers of PLP-Lys56 (internal aldimine). The Schiff base nitrogen is not protonated in (**bi**). (**ci**) External aldimine with L-serine. (**cii**) The 'quinonoid' intermediate in which the Cα loses a proton and becomes planar. The Cα can acquire a proton from Ser 84 to give the D-serine adduct (**ciii**), or the serine hydroxyl can be protonated and lost as a water. (**ciii**) External aldimine with D-serine. MarvinSketch 20.20 (https://www.chemaxon.com) was used to draw chemical structures and predict protonation and tautomeric states.

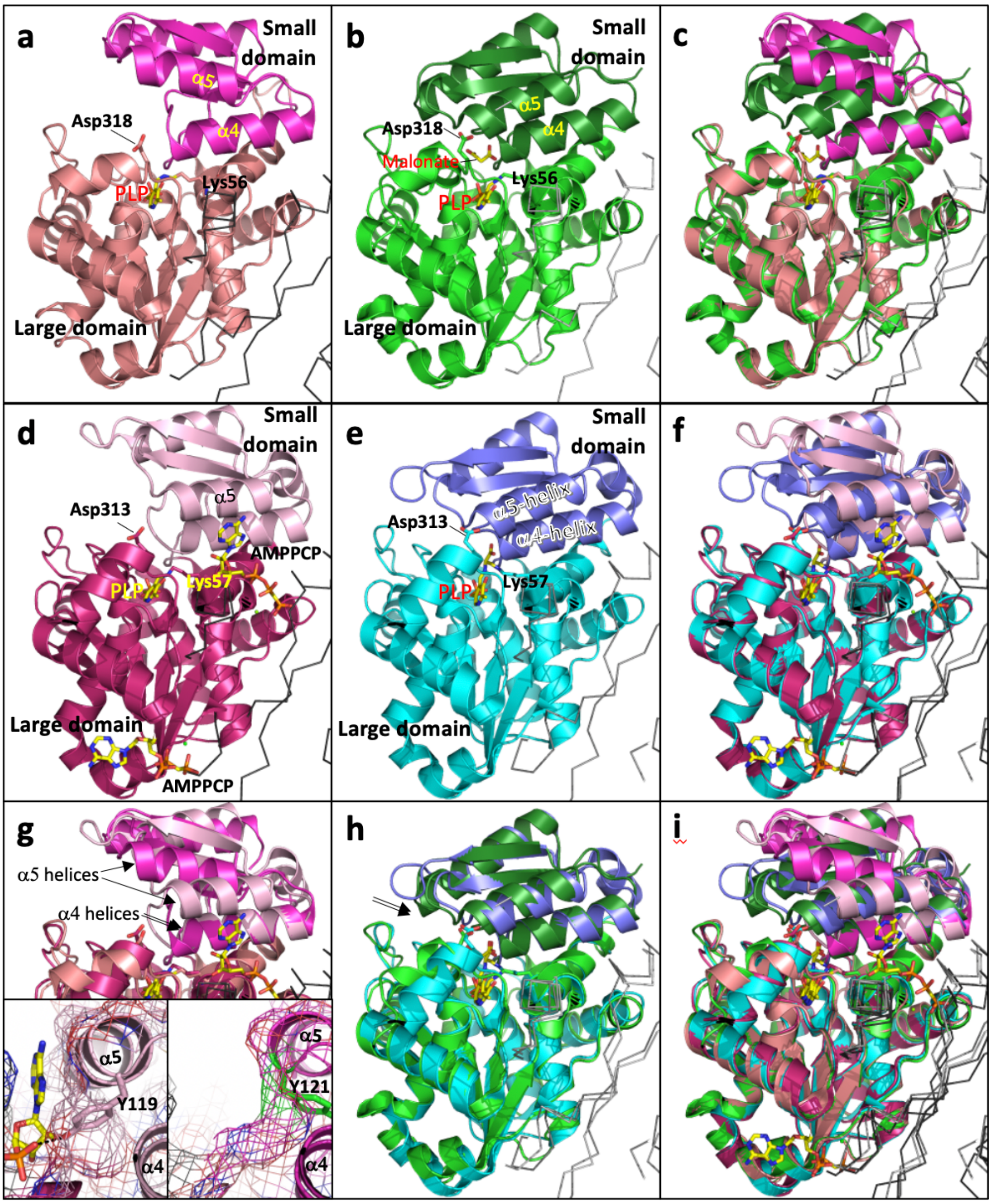

Supplementary Figure 2. Comparison of 'open' and 'closed' structures of human and yeast SR. (**a**) The 'open' 1.89 Å structure of *h*SR (pdb code: 6SLH). The view is from the second subunit in the dimer (Cα ribbon, dark grey). (**b**) The 'closed' 1.5 Å structure of *h*SR with PLP and malonate/MAL (pdb code: 3L6B). The N-terminus of the α4-helix interacts with a malonate carboxylic acid and the N-terminus of the α5-helix interacts with Asp 318 from the conserved SGGNV**D** sequence motif. (**c**) Superposition of (**a**) and (**b**) on large domains. (**d**) The 'open' 1.9 Å yeast structure containing AMPPCP (pdb code: 1WTC). The adenine ring of AMPPCP is close to the α4 and α5 helices. (**e**) The 'closed' 1.7 Å yeast structure (pdb code: 2ZPU) with PDD (N-5'-phosphopyridoxyl)-D-alanine) covalently attached to Lys 57. (**f**) Superposition of (**d**) and (**e**) on large domains. (**g**) Superposition of (**a**) and (**d**) on large domains. Note the large relative shift of the α5 helices, while α4 helices are in the 'same' position. Insets at bottom of panel **g** show how different positions of α5 helices gives an ATP binding pocket in the yeast apo structure (lower left), but not in the human. (**h**) Superposition of (**b**) and (**e**) on large domains. (**i**) Superposition of (**a**), (**b**), (**d**) and (**e**) on large domains.

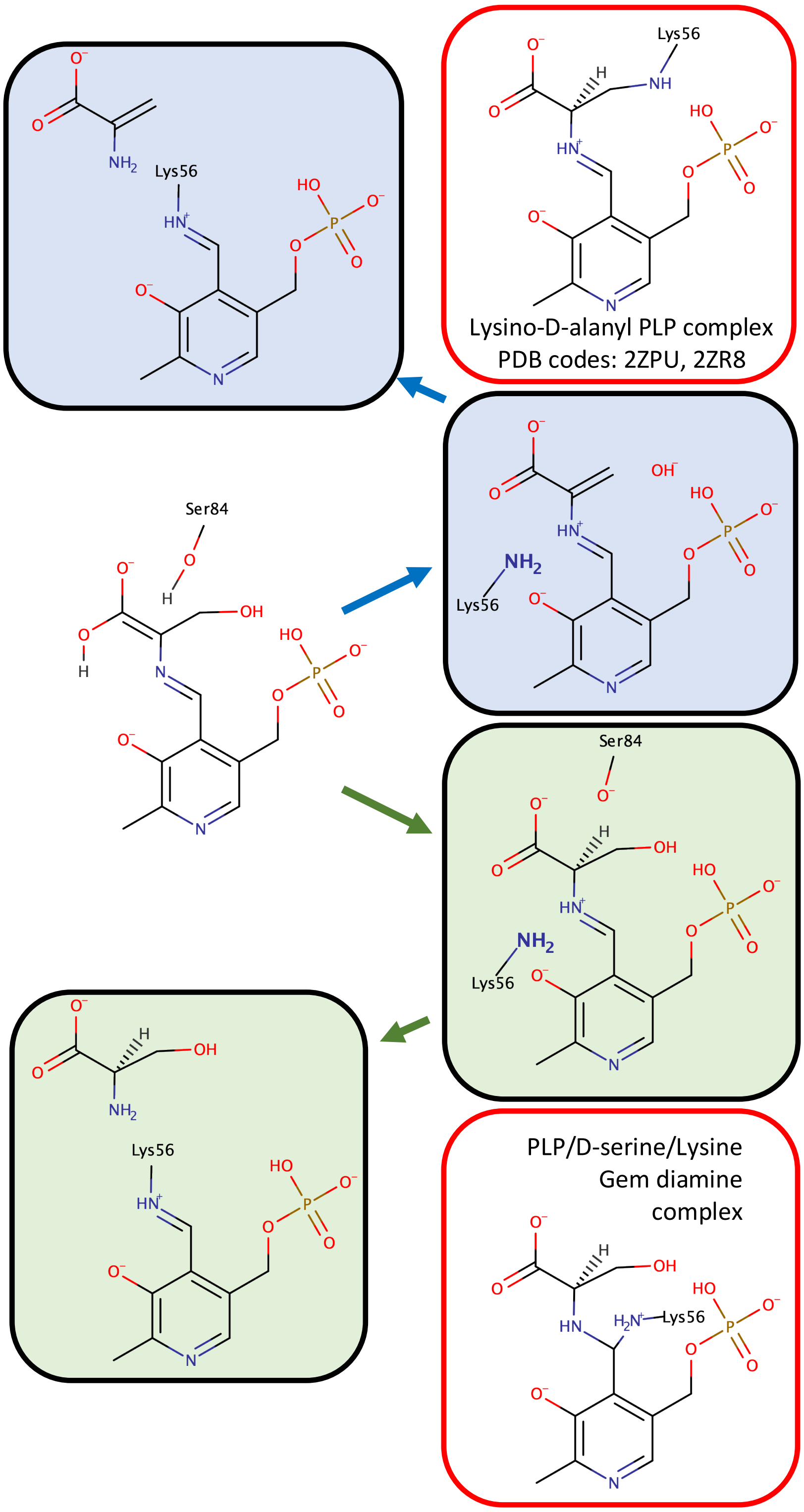

Supplementary Figure 3. **Serine racemase chemistry and lysino-d-alanyl and gem-diamine structures**. The reaction products of SR are normally aminoacrylate (blue pathway) and D-serine (green pathway)[33]. At the end of the catalytic cycle the catalytic lysine normally replaces the external aldimine with an internal aldimine. However, with *S. pombe* SR, lysino-D-alaynyl structures were observed and recently with a thermophilic serine hydroxymethyltransferase, a gem diamine structure has been observed (pdb code: 6TI4). MarvinSketch 20.20 (https://www.chemaxon.com) was used to draw chemical structures and predict protonation and tautomeric states.

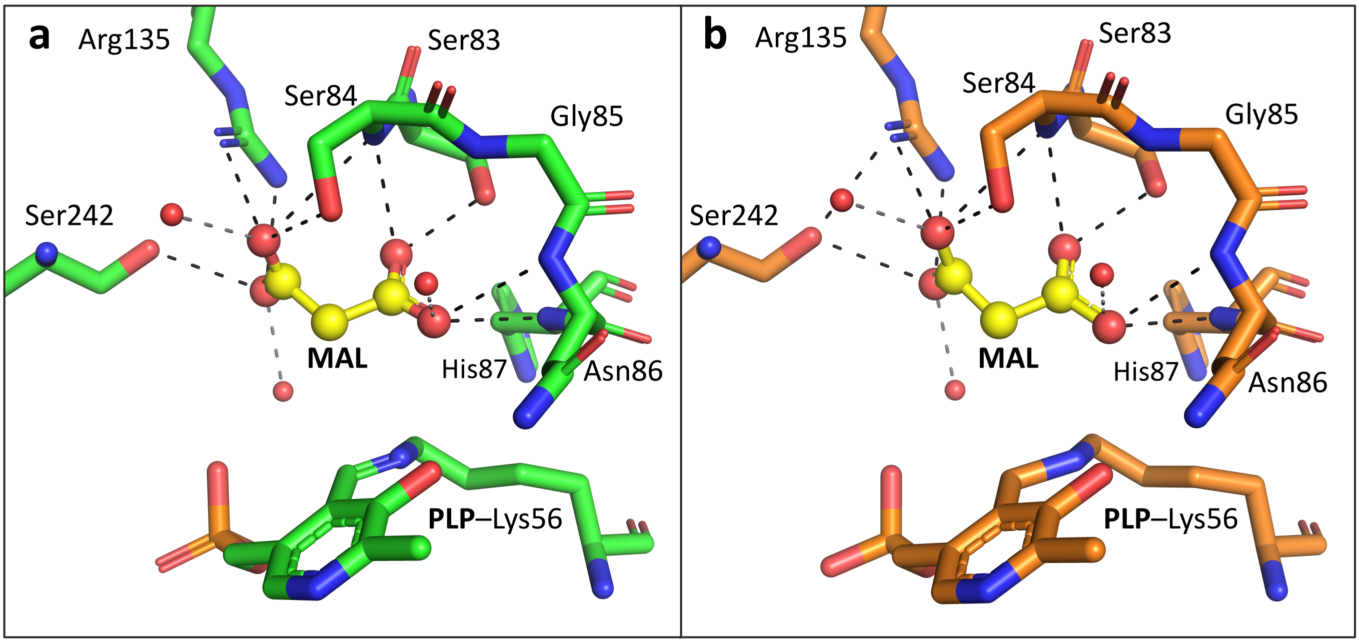

Supplementary Figure 4. Malonate binding sites in human SR crystal structures (6ZSP and 3L6B). (**a**) Malonate binding site in 1.6 Å ATP-bound structure (pdb code: 6ZSP). Each of the four oxygens (red atoms) of malonate (yellow carbons) is within H-bonding distance (black dashed lines) of two or three H-bond donors. These include: 5 NHs (blue atoms) from Arg 135 side-chain; main-chain NHs of Ser 84, Asn 86, and His 87; 3 OHs from serine residues 83, 84, and 242; and 3 waters. PLP is covalently attached to Lys 56. (**b**) Malonate binding site of previous SR structure without ATP (pdb code: 3L6B)[3]. The position of malonate and PLP and the organisation of the binding site is virtually identical to that shown in (**a**).

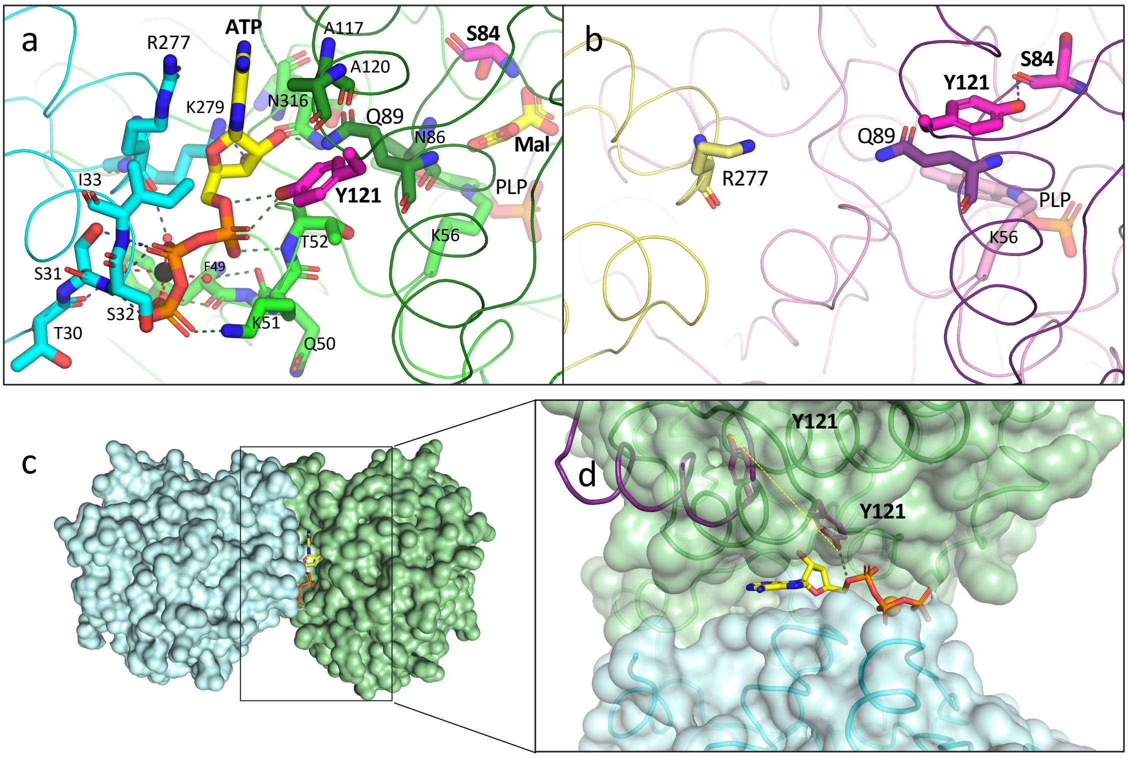

Supplementary Figure 5. Tyr121 can form hydrogen bonds to ATP or main-chain carbonyl of Ser 84. (**a**) Close-up of ATP binding site from 1.6 Å ATP/malonate-bound structure (pdb code: 6ZSP) showing ATP (yellow carbons) H-bonded to residues in the small domain (dark green) and large domain (light green) of subunit A in human SR. Tyr121 forms H-bonds to the α-phosphate and Gln 89 (side chain in two conformers) forms H-bonds with oxygens from the ribose. Two of the waters (small red spheres) that coordinate the ATP-associated Mg^{2+} ion (black sphere) also form H-bonds with the large domain of the second subunit in the dimer (cyan carbons). (**b**) Equivalent view of new SR holoenzyme structure (pdb code: 6ZUJ). Note the difference in orientation of the side chain of Tyr 121, which is now H-bonded to Ser 84. R277 side-chain is disordered (not shown). (**c**) Surface representation of ATP/malonate-bound structure in similar orientation to (**a**). (**d**) Tyr 121 is oriented towards the ATP site and contributes to the formation of a tightly-fitting ATP binding pocket. The surface is semitransparent, and the position adopted by Tyr 121 at the C-terminal end of the α5 helix (PDCKKLAIQAY) in the SR holoenzyme structure is also shown (purple ribbon, tyrosine as stick). The two positions occupied by the terminal hydroxyls of Tyr121 in the two structures are denoted by a dotted yellow line representing a distance of 10 Å.

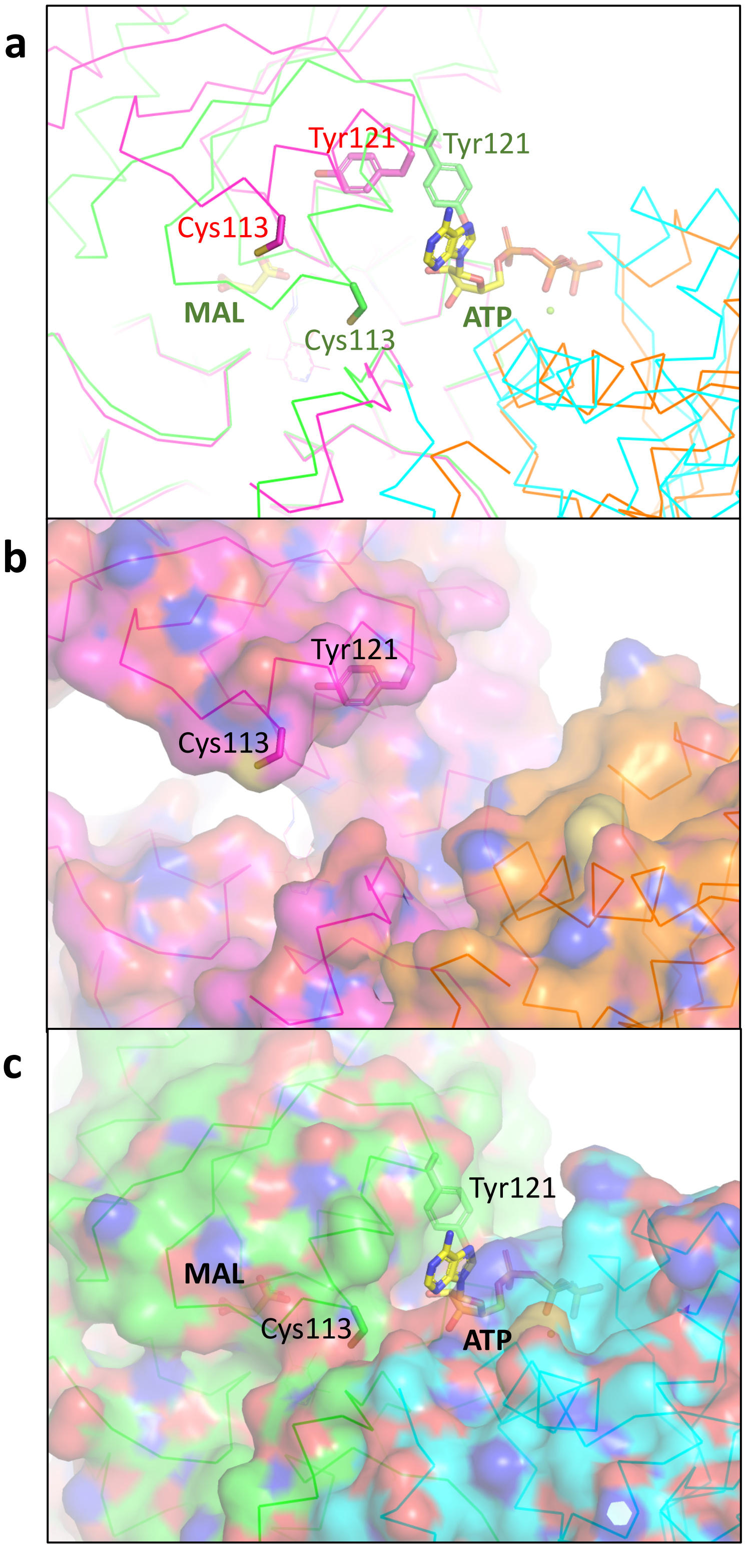

Supplementary Figure 6. The side-chain of Cys 113 is accessible in the 'open' human SR structure but is buried in the 'closed' structure. (**a**) Comparison of 'open' and 'closed' structures (Ca traces; ATP, malonate (MAL), and side-chains of Cys 113 and Tyr 121 shown as sticks). (**b**) 'Open' structure as in (a) with semitransparent surface shown – note yellow surface area corresponding to SG of Cys113 (**c**) 'Closed' structure as in (a) with semi-transparent surface shown. The SG of Cys 113 is buried.

```
SRR_HUMAN    ------------MCAQYCISFADVEKAHINIRDSIHLTPVLTSSILNQLTGRNLFFKCELF 49
SRR_MOUSE    ------------MCAQYCISFADVEKAHINIQDSIHLTPVLTSSILNQIAGRNLFFKCELF 49
SRR_RAT      ------------MCAQYCISFADVEKAHLNIQDSVHLTPVLTSSILNQIAGRNLFFKCELF 49
SRR_SCHPO    -----------MSDNLVLPTYDDVASASERIKKFANKTPVLTSSTVNKEFVAEVFFKCENF 50
F5CAQ9_MAIZE MGSKDGTGDISEAQGYAADIDSIREAQARIAPYVHRTPVMSSTSIDAMVGKKLFFKCECF 60
                                  .: .*  .*    : ***::*: ::     ::***** *

SRR_HUMAN    QKTGSFKIRGALNAVRSLVPDALERKPKAVVTHSSGNHGQALTYAAKLEGIPAYIVVPQT 109
SRR_MOUSE    QKTGSFKIRGALNAIRGLIPDTPEEKPKAVVTHSSGNHGQALTYAAKLEGIPAYIVVPQT 109
SRR_RAT      QKTGSFKIRGALNAIRGLIPDTLEGKPKAVVTHSSGNHGQALTYAAKLEGIPAYIVVPQT 109
SRR_SCHPO    QKMGAFKFRGALNALSQLNE---AQRKAGVLTFSSGNHAQAIALSAKILGIPAKIIMPLD 107
F5CAQ9_MAIZE QKAGAFKIRGASNSIFALDD---EQVSKGVVTHSSGNHAAAVALAAKLRGIPAHIVIPRN 117
             ** *:**:*** *::  *          .*:*.*****. *:: :**: **** *::*

SRR_HUMAN    APDCKKLAIQAYGASIVYCEPSDESRENVAKRVTEETEGIMVHPNQEPAVIAGQGTIALE 169
SRR_MOUSE    APNCKKLAIQAYGASIVYCDPSDESREKVTQRIMQETEGILVHPNQEPAVIAGQGTIALE 169
SRR_RAT      APNCKKLAIQAYGASIVYSEPSDESRENVAQRIIQETEGILVHPNQEPAVIAGQGTIALE 169
SRR_SCHPO    APEAKVAATKGYGGQVIMYDRYKDDREKMAKEISEREGLTIIPPYDHPHVLAGQGTAAKE 167
F5CAQ9_MAIZE APASKVENVKCYGGHIIWSDASIESREYVSKRVQEETGAVLIHPINSKYTISGQGTVSLE 177
             ** .*    : **. ::  :   :.** :::.: :.    :: * :   .::**** : *

SRR_HUMAN    VLNQVPLVDALVVPVGGGGMLAGIAITVKALKPSVKVYAAEPSNADDCYQSKLKGKLMPN 229
SRR_MOUSE    VLNQVPLVDALVVPVGGGGMVAGIAITIKALKPSVKVYAAEPSNADDCYQSKLKGELTPN 229
SRR_RAT      VLNQVPLVDALVVPVGGGGMVAGIAITIKTLKPSVKVYAAEPSNADDCYQSKLKGELTPN 229
SRR_SCHPO    LFEEVGPLDALFVCLGGGGLLSGSALAARHFAPNCEVYGVEPEAGNDGQQSFRKGSIVH- 226
F5CAQ9_MAIZE LLEQVPEIDTIIVPISGGGLISGVALAAKAINPSIRILAAEPKGADDSAQSKAAGKIIT- 236
             ::::*  :*::.* :.***:::* *:: : : *. .: ..**. .:*  **   *.:

SRR_HUMAN    LYPPETIADGVK-SSIGLNTWPIIRDLVDDIFTVTEDEIKCATQLVWERMKLLIEPTAGV 288
SRR_MOUSE    LHPPETIADGVK-SSIGLNTWPIIRDLVDDVFTVTEDEIKYATQLVWGRMKLLIEPTAGV 288
SRR_RAT      LHPPETIADGVK-SSIGLNTWPIIRDLVDDVFTVTEDEIKYATQLVWERMKLLIEPTAGV 288
SRR_SCHPO    IDTPKTIADGAQTQHLGNYTFSIIKEKVDDILTVSDEELIDCLKFYAARMKIVVEPTGCL 286
F5CAQ9_MAIZE LPSTNTIADGLR-AFLGDLTWPVVRDLVDDVIVVDDTAIVDAMKMCYEILKVAVEPSGAI 295
             :   :***** :   :*  *: :::: ***::.* :  :  . ::    :*: :**:. :

SRR_HUMAN    GVAAVLSQHFQTVSP--EVKNICIVLSGGNVDLTSSITWVKQAERPASYQSVSV 340
SRR_MOUSE    ALAAVLSQHFQTVSP--EVKNVCIVLSGGNVDLT-SLNWVGQAERPAPYQTVSV 339
SRR_RAT      GLAAVLSQHFQTVSP--EVKNICIVLSGGNVDLT-SLSWVKQAERPAP------ 333
SRR_SCHPO    SFAAARAMKE-----KLKNKRIGIIISGGNVDIERYAHFLSQ------------ 323
F5CAQ9_MAIZE GLAAALSDEFKQSSAWHESSKIGIIVSGGNVDLGTL--WQSMYKH--------- 338
             ..**. : .        : ..: *::******:     :
```

Supplementary Figure 7. Serine racemase sequence alignment. SR amino acid sequences from *H. sapiens* (SRR_HUMAN), *M. musculus* (SRR_MOUSE), *R. norvegicus* (SRR_RAT), *S. pombe* (SRR_SCHPO) and *Z. mays* (F5CAQ9_MAIZE) were aligned with ClustalO using sequences from UniProt[34]. The small domain of *h*SR (residues 78–155) is underlined. Residues (S84, G88, Q89, T92, I104, A117, I118) that are within 3.8 Å of the side chain of Tyr 121 (Y121) in the 'open' structure (pdb code: 6ZUJ) are highlighted in red on the *h*SR (SRR_HUMAN) sequence. Note that G88 and T92 are alanines in the *S. pombe* and *Z. Mays* sequences. Conserved residues are indicated by * underneath alignment. Highlighted residues are C113 (the nitrosylation site in SRR_MOUSE[28]) and T71 and T227 (the phosphorylation sites in SRR_MOUSE[35]).

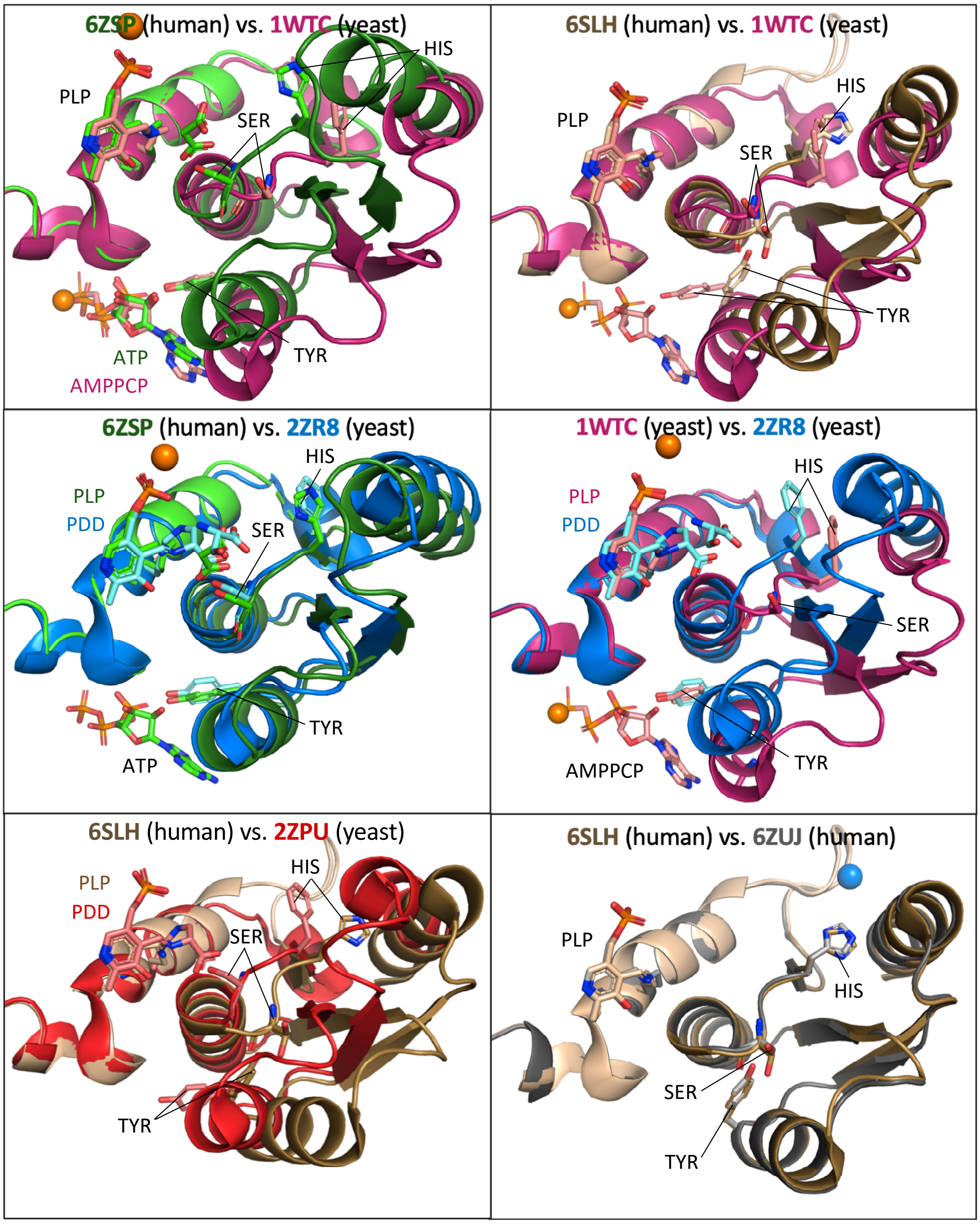

Supplementary Figure 8. Superpositions of human *holo* and ATP/malonate-bound SR with yeast orthologues. The displayed structures are: **6ZSP** (green; human malonate-bound with ATP); **1WTC** (magenta; yeast *holo* with ATP analogue, AMPPCP); **6SLH** (small domain brown, side-chains + large domain light pink; human *holo*); **2ZR8** (blue; with both L-Ser and modified PLP – called PDD see supplementary Table 2); **2ZPU** (red; yeast *holo*); **6ZUJ** (grey, new XChem human *holo*). The residues in stick are TYR (Tyr 121 in human, Tyr 119 in yeast), SER (Ser 84 in human, Ser 82 in yeast) and HIS (His 82 in human, His 80 in yeast).

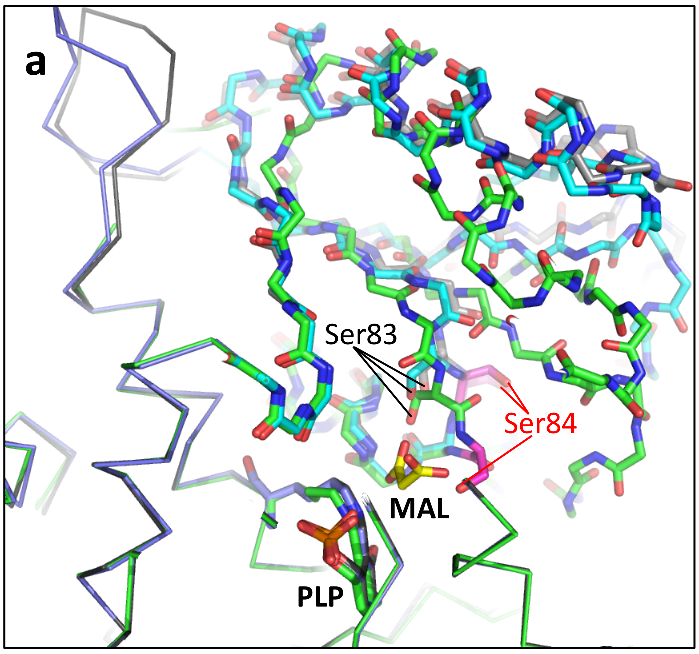

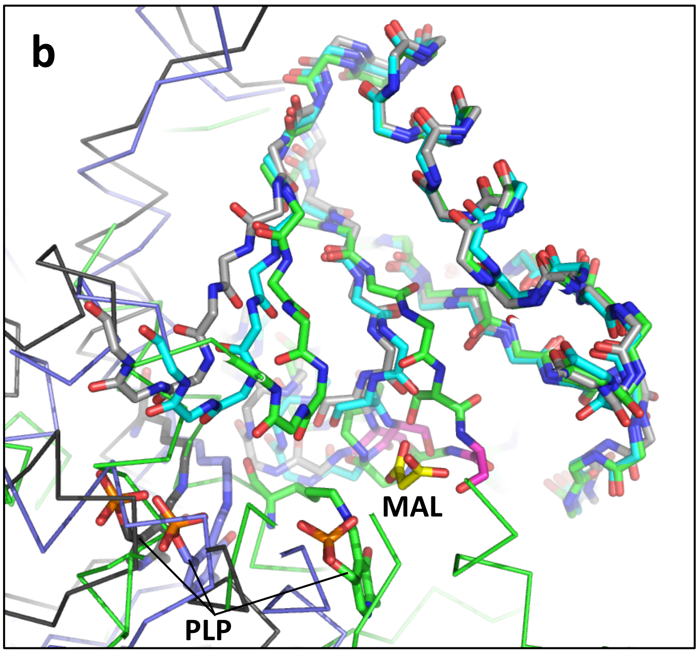

Supplementary Figure 9. Superpositions of ‘closed’ SR ATP/malonate-bound structure with ‘open’ SR subunits A and C. (**a**) The ‘closed’ *h*SR (green carbons) complex with malonate/MAL (stick; yellow carbons) and ATP (not visible) is shown with large domain as Cα ribbon and small domain as backbone sticks. The side-chain of Ser 84 (stick magenta carbons) is close to the malonate. The PLP (stick; green carbons) is covalently attached to Lys 56. Superposed on Mal/ATP large domain are the A subunit of an XChem structure (blue/cyan carbons, compound **13**-x0430 – compound not observed in A subunit) and the C subunit of an XChem structure (grey carbons, compound **1**-x0495 - compound not seen in C subunit). Side-chain hydroxyls of Ser 83 and 84 are arrowed. (**b**) The closed *h*SR (green carbons) complex with malonate/MAL (stick; yellow carbons) and ATP (not visible) as in (a). The XChem A and C subunits are superposed based on residues 78–81 and 101–148 of the small domain (residues 77–155). Note large relative shifts of PLP and large domain.

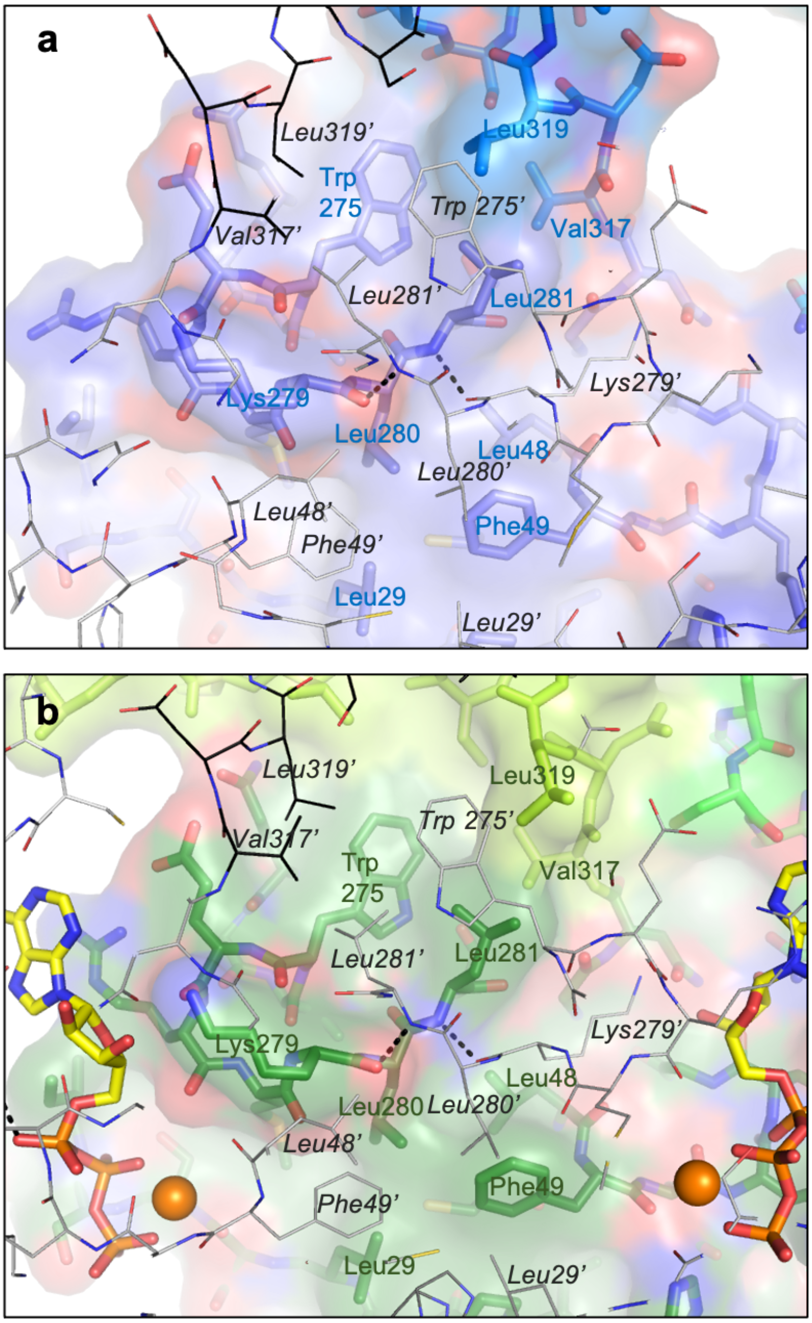

Supplementary Figure 10. The hydrophobic core of the dimer interface in 'open' and 'closed' hSR structures. (**a**) The AC dimer interface from an 'open' XChem structure (pdb code: 6ZUJ). The A subunit (stick; blue carbons) is shown with a semitransparent surface. The C subunit (line representation; grey carbons) is nearer the viewer. Residues are labelled with blue letters for the A subunit and black letters for the C subunit. Intersubunit hydrogen bonds between CO of Lys 279 to the NH of Leu 281' and from the NH of Leu 281 to CO of Lys 279' are indicated by dotted lines. (**b**) The AB dimer interface from the 'closed' malonate/ATP structure (pdb code: 6ZSP). The A subunit (stick; green carbons) is shown with a semitransparent surface. The B subunit (line representation; grey carbons) is nearer the viewer. ATPs (stick, yellow carbons) and magnesium ions (orange spheres) are also present.

Supplementary Figure 11. ATP dependent activity data on Wild-type, Y121A, Y121F and Y121G mutants

The activity of the the wild-type and the three Y121 mutants: Y121F, Y121A and Y121G, was measured at six ATP concentrations (1mM, 0.5mM, 0.25mM, 0.125mM, 0.062mM and 0.031mM) - see Methods for details. Error bars show standard error.

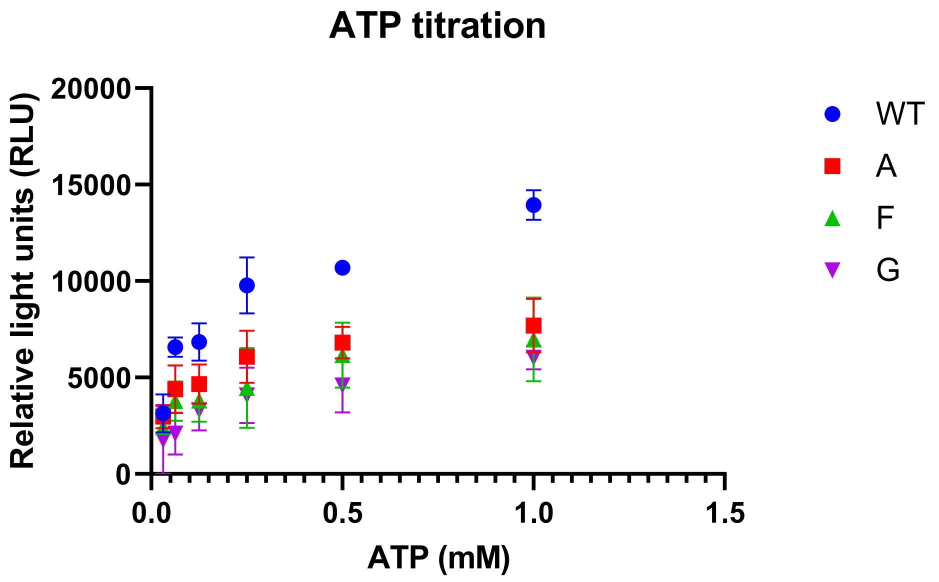

Supplementary Figure 12. Inhibition data on Wild-type, Y121A, Y121F and Y121G mutants of hSR. The compounds were all assayed in 16.7% DMSO. The maximum compound concentration used in the assay was 16.7mM. Compounds 3-x0458, 4-x0482 and 13-x0430 showed 100% inhibition at maximum concentration - and therefore have accurate IC50(s) - see supplementary table 4. Assays were performed at least 3 times to give accurate estimates of errors, below are shown single assays; examples of data from which IC50's in supplementary table 4 were calculated (compound concentrations used were 16.7mM, 5.55mM, 1.85mM, 0.62mM, 0.21mM, 0.069mM, 0.023mM and 0.0077mM). Error bars show standard error.

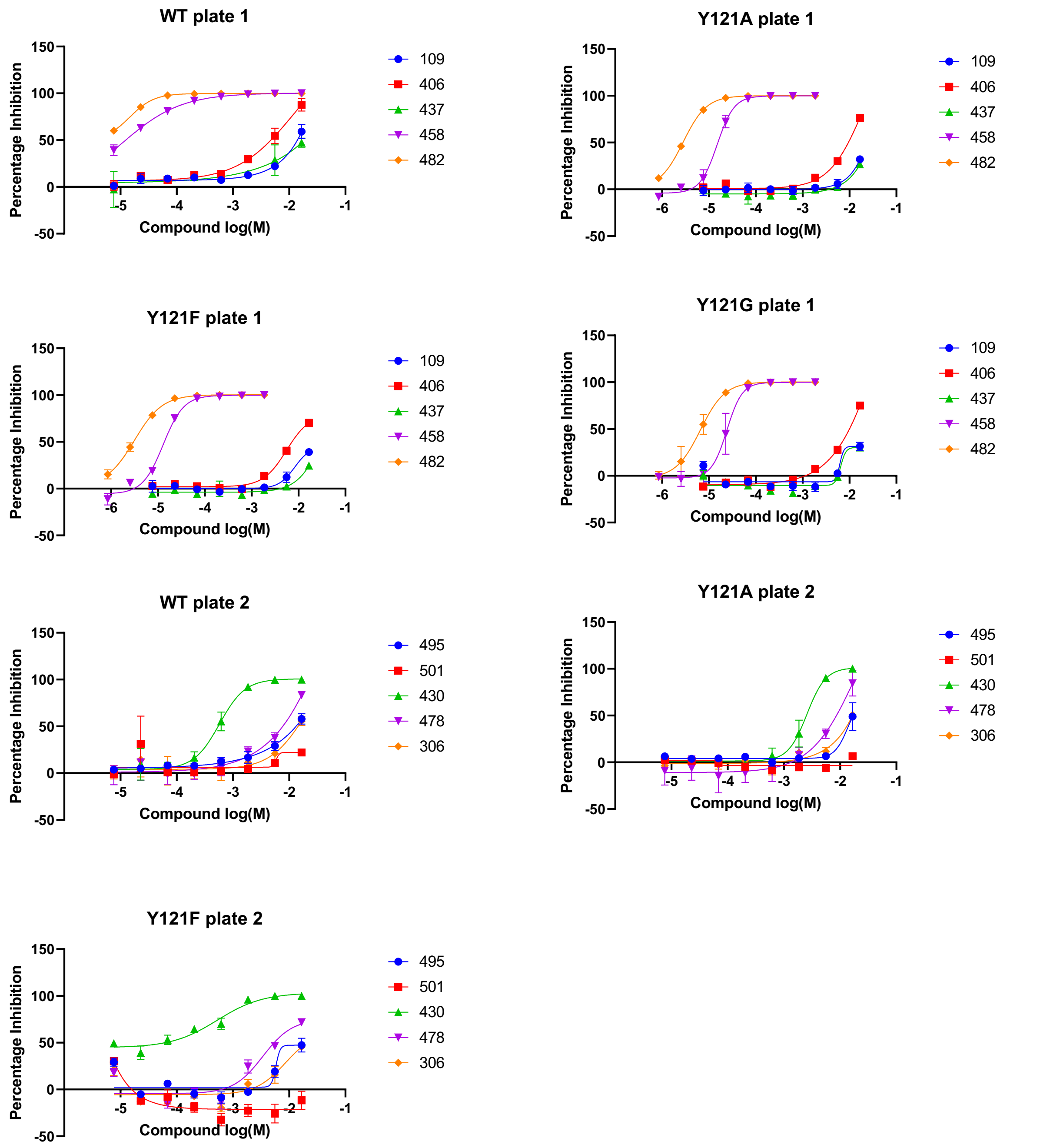

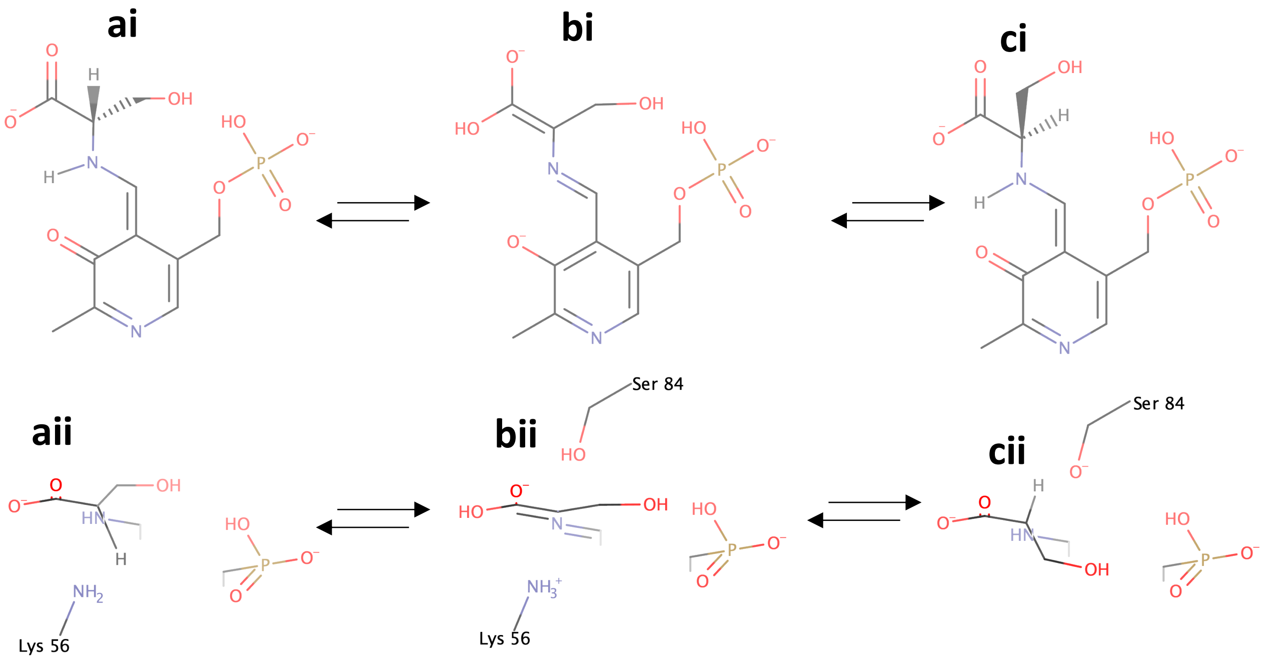

Supplementary Figure 13. Roles of lysine 56 and serine 84 in proton exchange in human SR.

(**ai, aii**) Orthogonal view of an L-serine PLP external aldimine. The view in (**aii**) is looking down from above (**ai**). Only the uppermost atoms of (**ai**) are shown in (**aii**). Lys 56 extracts the Cα proton between (**ai/aii**) and (**bi/bii**) or donates the proton in the reverse process. (**bi/bii**) Orthogonal views of a planar intermediate form in which the Cα of serine has no hydrogen. (**ci/cii**) Orthogonal view of a D-serine PLP external aldimine. Ser 84 donates (**b** to **c**) or extracts (**c** to **b**) the Cα proton. MarvinSketch 20.20 (https://www.chemaxon.com) was used to draw chemical structures and predict protonation and tautomeric states.

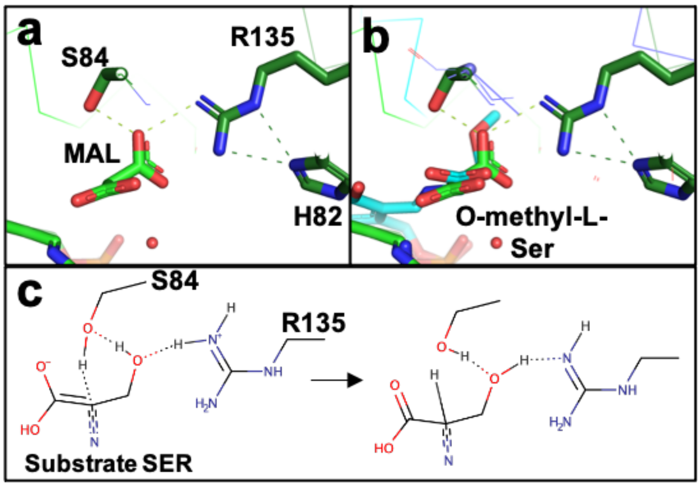

Supplementary Figure 14. Does Arginine 135 protonate Ser 84 via the side-chain of the substrate serine? (**a**) A hydrogen bond network between Ser 84, malonate (MAL), Arg 135 and His 82 in the 1.6 Å hSR structure (pdb code: 6ZSP). (**b**) The structure of SDH in complex with O-methyl-serine (pdb code: 1PWH) is shown superposed on the 1.6 Å malonate/ATP structure. (**c**) Proposed mechanism demonstrating how Arg 135 could protonate the hydroxyl of the substrate serine as it in turn protonates Ser 84. The mechanism suggests that Arg 135 could protonate the hydroxyl of the substrate serine; the substrate serine protonates Ser 84, which in turn protonates the Cα of the substrate. Arg 135 is conserved in serine racemase sequences but is not present in serine dehydratases (see Supplementary Fig. 15).

```
SRR_HUMAN    -----------MCAQYCISFADVEKAHINIRDSIHLTPVLTSSILNQLTGRNLFFKCELF 49
SRR_RAT      -----------MCAQYCISFADVEKAHLNIQDSVHLTPVLTSSILNQIAGRNLFFKCELF 49
SRR_SCHPO    ----------MSDNLVLPTYDDVASASERIKKFANKTPVLTSSTVNKEFVAEVFFKCENF 50
F5CAQ9_MAIZE MGSKDGTGDISEAQGYAADIDSIREAQARIAPYVHRTPVMSSTSIDAMVGKKLFFKCECF 60
SDHL_HUMAN   --------------------------MMSGEPLHVKTPIRDSMALSKMAGTSVYLKMDSA 34
SDHL_RAT     --------------------------MAAQESLHVKTPLRDSMALSKVAGTSVFLKMDSS 34
                                                 **:  *  :.     .:::* :
SRR_HUMAN    QKTGSFKIRGALNAVRSLVPDALERKPKAVVTHSSGNHGQALTYAAKLEGIPAYIVVPQT 109
SRR_RAT      QKTGSFKIRGALNAIRGLIPDTLEGKPKAVVTHSSGNHGQALTYAAKLEGIPAYIVVPQT 109
SRR_SCHPO    QKMGAFKFRGALNALSQLNE---AQRKAGVLTFSSGNHAQAIALSAKILGIPAKIIMPLD 107
F5CAQ9_MAIZE QKAGAFKIRGASNSIFALDD---EQVSKGVVTHSSGNHAAAVALAAKLRGIPAHIVIPRN 117
SDHL_HUMAN   QPSGSFKIRGIGHFCKRW----AKQGCAHFVCSSAGNAGMAAAYAARQLGVPATIVVPST 90
SDHL_RAT     QPSGSFKIRGIGHLCKMK----AKQGCKHFVCSSAGNAGMATAYAARRLGLPATIVVPST 90
             *  *:**:**  :                .:  *:** . * : :*:  *:** *::*
SRR_HUMAN    APDCKKLAIQAYGASIVYCEPSDESRENVAKRV-TEETEGIMVHPNQEPAVIAGQGTIAL 168
SRR_RAT      APNCKKLAIQAYGASIVYSEPSDESRENVAQRI-IQETEGILVHPNQEPAVIAGQGTIAL 168
SRR_SCHPO    APEAKVAATKGYGGQVIMYDRYKDDREKMAKEI-SEREGLTIIPPYDHPHVLAGQGTAAK 166
F5CAQ9_MAIZE APASKVENVKCYGGHIIWSDASIESREYVSKRV-QEETGAVLIHPINSKYTISGQGTVSL 176
SDHL_HUMAN   TPALTIERLKNEGATVKVVGELLDEAFELAKALAKNNPGWVYIPPFDDPLIWEGHASIVK 150
SDHL_RAT     TPALTIERLKNEGATVEVVGEMLDEAIQLAKALEKNNPGWVYISPFDDPLIWEGHTSLVK 150
             :*  .    :  *. :       :.   ::: :  :.     : * :      *: :
SRR_HUMAN    EVLNQVPL-VDALVVPVGGGGMLAGIAITVKALK-PSVKVYAAEPSNADDCYQSKLKGKL 226
SRR_RAT      EVLNQVPL-VDALVVPVGGGGMVAGIAITIKTLK-PSVKVYAAEPSNADDCYQSKLKGEL 226
SRR_SCHPO    ELFEEVGP-LDALFVCLGGGGLLSGSALAARHFA-PNCEVYGVEPEAGNDGQQSFRKGSI 224
F5CAQ9_MAIZE ELLEQVPE-IDTIIVPISGGGLISGVALAAKAIN-PSIRILAAEPKGADDSAQSKAAGKI 234
SDHL_HUMAN   ELKETLWEKPGAIALSVGGGGLLCGVVQGLQEVGWGDVPVIAMETFGAHSFHAATTAGKL 210
SDHL_RAT     ELKETLSAKPGAIVLSVGGGGLLCGVVQGLREVGWEDVPIIAMETFGAHSFHAAVKEGKL 210
             *: :    .:: : :.***::.* .   : .   .  : . *   ...   :   *.:
SRR_HUMAN    MPNLYPPETIADGVK-SSIGLNTWPIIRDLVDDIFTVTEDEIKCATQLVWERMKLLIEPT 285
SRR_RAT      TPNLHPPETIADGVK-SSIGLNTWPIIRDLVDDVFTVTEDEIKYATQLVWERMKLLIEPT 285
SRR_SCHPO    VH-IDTPKTIADGAQTQHLGNYTFSIIKEKVDDILTVSDEELIDCLKFYAARMKIVVEPT 283
F5CAQ9_MAIZE IT-LPSTNTIADGLR-AFLGDLTWPVVRDLVDDVIVVDDTAIVDAMKMCYEILKVAVEPS 292
SDHL_HUMAN   VS-LPKITSVAKALGVKTVGAQALKLFQEHPIFSEVISDQEAVAAIEKFVDDEKILVEPA 269
SDHL_RAT     VT-LPKITSVAKALGVNTVGAQTLKLFYEHPIFSEVISDQEAVTAIEKFVDDEKILVEPA 269
                :    ::*..     :*  :  :. :      .: :     . :      *: :**:
SRR_HUMAN    AGVGVAAVLSQHFQTVSP-------EVKNICIVLSGGNVDLTSSITWVKQAERPASYQSVSV 340
SRR_RAT      AGVGLAAVLSQHFQTVSP-------EVKNICIVLSGGNVDLT-SLSWVKQAERPAP------ 333
SRR_SCHPO    GCLSFAAARAMKE-----KL-----KNKRIGIIISGGNVDIERYAHFLSQ------------ 323
F5CAQ9_MAIZE GAIGLAAALSDEFKQSSAWH-----ESSKIGIIVSGGNVDLGTL--WQSMYKH--------- 338
SDHL_HUMAN   CGAALAAVYSHVIQKLQLEGNLRTPLPSLVVIVCGGSNISLAQLRALKEQLGMTNRLPK--- 328
SDHL_RAT     CGAALAAVYSGVVCRLQAEGRLQTPLASLVVIVCGGSNISLAQLQALKAQLGLNELLK---- 327
                ..**. :                 . : *: .*.*:.:
```

Supplementary Figure 15. Serine dehydratase and serine racemase sequence alignment. SR amino acid sequences are as described in Supplementary Fig. 7. Structures for all sequences are available in the PDB (see Supplementary Table 2). Serine dehydratase (SDH) sequences for *H. sapiens* (SDHL_HUMAN) and *R. norvegicus* (SDHL_RAT) (isoform 2) were obtained from UniProt[34]. Conserved residues are indicated by * underneath alignment. Residues highlighted in red on the *h*SR sequence (T52, K56, H82, S84, Q89, Y121, R135, E136). Note the conservation of S83, G85, and N86.

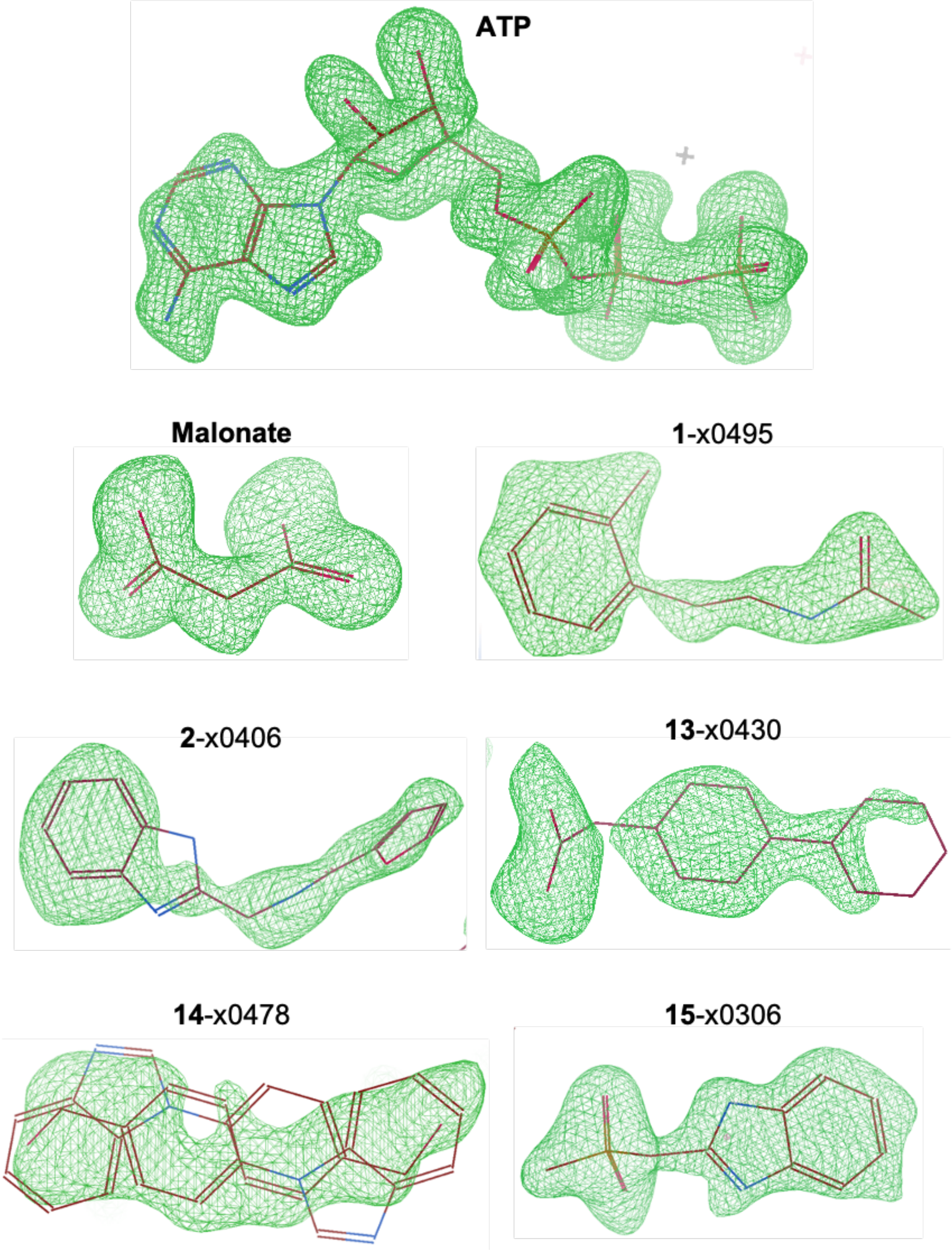

Supplementary Figure 16. OMIT maps of ATP, malonate, and bound XChem ligands. Fo-Fc electron density (green mesh) contoured to 3 σ showing evidence for bound ligand. OMIT maps were generated by removing the ligand from the structure and running five cycles of refinement in REFMAC. Shown here are ATP and malonate (pdb code: 6ZSP), **1**-x0495 (7NBG), **2**-x0406 (7NBH), **13**-x0430 (7NBC), **14**-x0478 (7NBD), and **15**-x0306 (7NBF). Compound **14**-x0478 sits on the twofold axis of the dimer and observed electron density is due to two (2-fold related) equivalent binding modes. Occupancies range from 0.7–1.0 (2 x 0.5 for **14**-x0478).

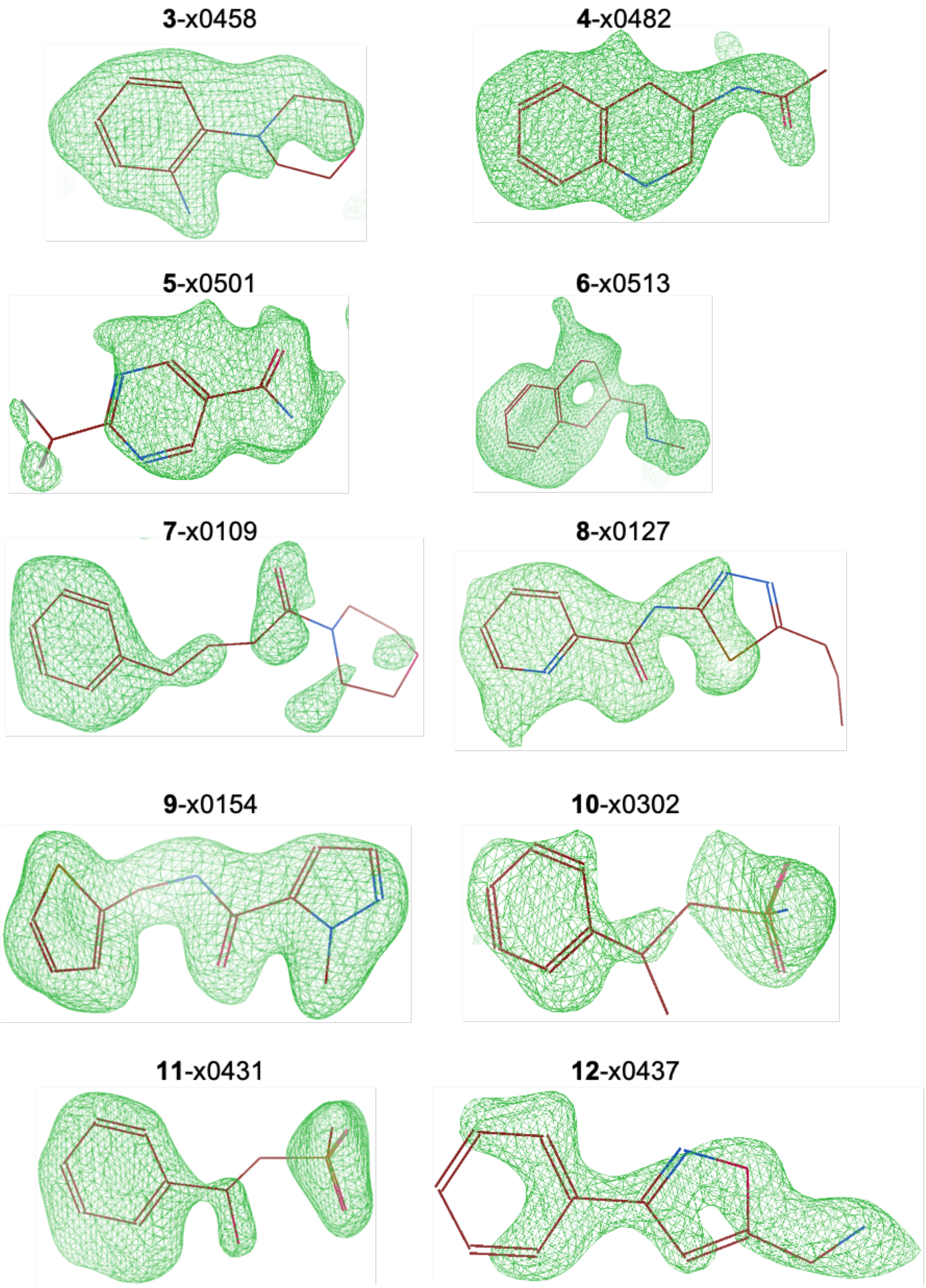

Supplementary Figure 17. OMIT maps of XChem ligands bound to SR. Fo-Fc electron density (green mesh) contoured to 3 σ showing evidence for bound XChem ligand. OMIT maps were generated by removing the ligand from the structure and running five cycles of refinement in REFMAC. Occupancies range from 0.7–1.0.

Supplementary Discussion.

The evolution and regulation of the activity of human serine racemase in the CNS.

In the CNS, SR can localise to specific regions in cells and its activity is tightly spatially and temporally regulated by a number of protein interactions[24-27,31,36-40] and post-translational modifications[28,35,36,41,42]. The emerging model suggests that astrocytes normally synthesize L-serine, which is then transported to neurons where D-serine is synthesised and utilized at synapses[24,40,43,44]. However, in the diseased or damaged brain, astrocytes can synthesize D-serine and the extra-synaptic activity of D-serine is neurotoxic[44]. SR interacts with postsynaptic proteins such as PICK1[26,45], GRIP[25,46], and PSD-95[31] to increase production of D-serine through unspecified structural mechanisms (see Supplementary Table 6 for a list of physiological modulators of SR). SR ubiquitination and degradation is tempered by its interaction with Golga3[27] and DISC1[32,40,47,48], the latter of which is linked to diminished D-serine and a schizophrenic phenotype when mutated. Understanding how SR activity is regulated in cells may facilitate optimisation of therapeutic inhibitors and activators of SR.

While it is clear that serine racemase and NMDA receptors play important roles in animals with a central nervous system, from *C. elegans* to man[49], more research remains to be done to understand exactly how SR activity is regulated *in vivo*.

How serine racemase activity evolved is also an intriguing question, one possibility is that SR evolved from a progenitor protein that had serine dehydratase activity but lacked racemisation activity. Human serine dehydratase (SDH), the closest human homologue of hSR with which it shares a common ancestor, is about 75-fold more active at elimination than human SR[9]. However, human SDH has no detectable elimination activity against D-serine. Racemisation is believed to have emerged as a side-reaction of the elimination function[50]. Comparison of structures of rat SDH with hSR (Fig. 8) suggested that Ser 84 is the crucial catalytic residue for racemisation[3], and this was confirmed by site-directed mutagenesis experiments in which the activity of a S84A mutant was determined[9]. The hSR S84A mutant had no detectable racemase activity against L-serine or D-serine. The S84A mutant also had no detectable elimination activity against D-serine while its activity against L-serine was comparable to wild-type[9]. However, the A65S mutant of SDH (Supplementary Fig. 15 for sequence alignment) had no detectable racemase activity[9] but it did acquire β-elimination activity against D-serine. Wild-type SDH only efficiently catalyses one reaction (the breakdown of L-serine into pyruvate and ammonia) of the four reactions on L- or D-serine catalysed by SR (L to D-Ser, D to L-Ser, L-Ser to pyruvate and ammonia, D-Ser to pyruvate and ammonia). The mutation A65S in SDH gives the enzyme some ability to catalyse the breakdown of D-serine to pyruvate and ammonia[9], supporting the generally accepted roles of Ser 84 and Lys 56 in hSR as the Si and Re-face acid/bases (Supplementary Fig. 13). However, if SR did indeed evolve from a progenitor of SDH, the acquisition of racemisation activity seems to require more than a simple acquisition of a serine at position 84 (=65 in rSD; Fig. 8).

In yeast *S. pombe* SR (*sp*SR), L-serine racemisation activity is over 20-fold lower than its elimination activity[5]. Mouse and human SR have comparable efficiencies for both reactions, with L-serine racemisation efficiency increased by over 10-fold relative to yeast SR while L-serine elimination is mostly unchanged[11,51]. Further, the *in vitro* rate of human SR racemisation is about 100-fold lower than that of alanine racemase, an archetypical amino acid racemase in bacteria[52], which supports the theory racemisation evolved later as a result of catalytic promiscuity[53]. Note, however, that *in vivo* activity is regulated by various interactions (see Supplementary Table 6) and could differ appreciably from *in vitro* activity.

The roles of Ser 84 and Lys 56 in abstracting or donating a proton from the central $C\alpha$ to convert L- to D-serine (or vice versa) is quite well understood (Supplementary Fig. 13). However, two alternate mechanisms have been proposed for the origin of the hydrogen in the β-elimination reaction. Smith *et al.*[3] state that the elimination reaction requires removal of water with the hydroxyl group from the $C\beta$ of L-serine and hydrogen from the lysyl-NH_3 as leaving groups. Another mechanism proposed for SDH states that the phosphate group of PLP acts as a general acid to donate a proton to the leaving hydroxyl group of the serine[7]. A proposed elimination mechanism (Fig. 9d) has the phosphate indirectly protonating the leaving hydroxyl, via a water. Unexpectedly in a recent neutron study of a PLP-dependent enzyme[54] a deuterium was observed equidistant between the Schiff base and the C-terminal carboxylate of the substrate. How protons move in the catalytic cycles of PLP dependent enzymes is not fully understood. The structure of yeast SR with a lysino-D-alanyl residue (Fig. 8)[5] showed that the catalytic lysine can become covalently attached to the substrate $C\beta$ side-chain[5]. Yamauchi et al.[5] reported that incubation of mouse SR with L-serine caused an enzyme to form with a higher mass than the native enzyme, which may indicate the formation of the lysine-substrate complex even in mammalian SRs. The broad range of possible protonation and tautomeric states[55] of the substrate/PLP conjugate (Supplementary Fig. 1) suggests that further studies to experimentally determine hydrogen positions as the enzyme moves through the racemisation or elimination catalytic cycle would be of interest. Close inspection of our 1.6 Å malonate/ATP structure suggests that the acidity of Ser 84 may be enhanced by Arg 135 (Supplementary Fig. 14), consistent with the conservation of Arg 135 in serine racemase but not serine dehydratase (Supplementary Fig. 15). However, in 2017, Nelson et al. (36) proposed that Lys 114 might play an important role in enhancing the ability of Ser 84 to protonate the substrate and thus produce D-Serine. The proposed mechanism (Fig. 9c) adequately explains the protonation of a planar quinonoid, although other possible mechanisms exist (e.g. Supplementary Fig. 14). However, neither mechanism (Fig. 9c, Supplementary Fig. 14) provides a ready explanation for the role of Ser 84 in abstracting the proton from the Ca of a D-serine substrate (Supplementary Fig. 13).

The sensitivity of SR to ATP activation varies among the SR orthologues with a conserved tyrosine. In slime mould (*D. discoideum*) SR, Mg-ATP increases racemisation by 2.5-fold and elimination by 6-fold, indicating the presence of an ATP site[56]. Yeast SR is minimally sensitive to ATP as both reactions increase by less than 2-fold[4]. In human SR, L-

serine elimination is most profoundly affected, increasing by 31-fold upon addition of ATP, followed by D-serine elimination which increases by 4-fold and finally L-serine racemisation by almost 2-fold[57,58]. Thus *in vitro*, the L-serine elimination reaction is several times more efficient than the L-serine racemisation reaction[51,57,58]. SDH does not have a conserved tyrosine or an ATP site and is not regulated by nucleotides[9], suggesting ATP binding is coupled to SR's evolutionary divergence from dehydratases.

The role of the Tyr 121 flip appears to be most relevant for the mammalian enzyme, where the complexity of the nervous system necessitates stricter maintenance of D-serine homeostasis in neurons. SR is thought to alternately synthesise and degrade D-serine in brain regions lacking D-amino acid oxidase (DAO), the main degradative enzyme for D-amino acids[59] that is restricted to the cerebellum and brainstem. It has been reported that enhanced astrocytic D-serine synthesis underlies synaptic damage after traumatic brain injury[60] and that neurotoxic astrocytes synthesize SR in Alzheimer's disease[61]. SR mutants deficient in elimination activity accumulate D-serine[62], therefore the elimination function of mammalian SR is critical with respect to preventing D-serine related neuropathologies. Several small molecules and post-translational modifications reduce SR activity in an ATP-dependent manner, including NADH[30], the membrane-localised inhibitor PIP2[24], and S-nitrosylation[28]. The Tyr 121 flip may serve to regulate serine metabolism by preventing basal degradation or synthesis of D-serine when ATP has been displaced by external regulators. When bound, the position of ATP appears to restrict how the small domain moves relative to the large domain, which may explain why ATP enhances racemisation less potently than L-serine elimination.

Supplementary References

1 Koulouris, C. R., Bax, B. D., Atack, J. R. & Roe, S. M. Conformational flexibility within the small domain of human serine racemase. *Acta Crystallog. sect. F* **76**, doi:10.1107/S2053230X920001193 (2020).

2 Takahara, S. *et al.* Design, synthesis, and evaluation of novel inhibitors for wild-type human serine racemase. *Bioorganic & medicinal chemistry letters* **28**, 441-445, doi:10.1016/j.bmcl.2017.12.021 (2018).

3 Smith, M. A. *et al.* The structure of mammalian serine racemase: evidence for conformational changes upon inhibitor binding. *J Biol Chem* **285**, 12873-12881, doi:10.1074/jbc.M109.050062 (2010).

4 Goto, M. *et al.* Crystal structure of a homolog of mammalian serine racemase from Schizosaccharomyces pombe. *J Biol Chem* **284**, 25944-25952, doi:10.1074/jbc.M109.010470 (2009).

5 Yamauchi, T. *et al.* Serine racemase with catalytically active lysinoalanyl residue. *J Biochem* **145**, 421-424, doi:10.1093/jb/mvp010 (2009).

6 Zou, L. *et al.* Crystal structure of maize serine racemase with pyridoxal 5'-phosphate. *Acta crystallographica. Section F, Structural biology communications* **72**, 165-171, doi:10.1107/S2053230X16000960 (2016).

7 Yamada, T. *et al.* Crystal structure of serine dehydratase from rat liver. *Biochemistry* **42**, 12854-12865, doi:10.1021/bi035324p (2003).

8 Sun, L., Bartlam, M., Liu, Y., Pang, H. & Rao, Z. Crystal structure of the pyridoxal-5'-phosphate-dependent serine dehydratase from human liver. *Protein Sci* **14**, 791-798, doi:10.1110/ps.041179105 (2005).

9 Wang, C. Y., Ku, S. C., Lee, C. C. & Wang, A. H. Modulating the function of human serine racemase and human serine dehydratase by protein engineering. *Protein Eng Des Sel* **25**, 741-749, doi:10.1093/protein/gzs078 (2012).

10 Yamada, T. *et al.* A catalytic mechanism that explains a low catalytic activity of serine dehydratase like-1 from human cancer cells: crystal structure and site-directed mutagenesis studies. *Biochim Biophys Acta* **1780**, 809-818, doi:10.1016/j.bbagen.2008.01.020 (2008).

11 Strisovsky, K., Jiraskova, J., Mikulova, A., Rulisek, L. & Konvalinka, J. Dual substrate and reaction specificity in mouse serine racemase: identification of high-affinity dicarboxylate substrate and inhibitors and analysis of the beta-eliminase activity. *Biochemistry* **44**, 13091-13100, doi:10.1021/bi051201o (2005).

12 Panizzutti, R., De Miranda, J., Ribeiro, C. S., Engelender, S. & Wolosker, H. A new strategy to decrease N-methyl-D-aspartate (NMDA) receptor coactivation: inhibition of D-serine synthesis by converting serine racemase into an eliminase. *Proceedings of the National Academy of Sciences of the United States of America* **98**, 5294-5299, doi:10.1073/pnas.091002298 (2001).

13 Dunlop, D. S. & Neidle, A. Regulation of serine racemase activity by amino acids. *Brain Res Mol Brain Res* **133**, 208-214, doi:10.1016/j.molbrainres.2004.10.027 (2005).

14 Hoffman, H. E. *et al.* Hydroxamic acids as a novel family of serine racemase inhibitors: mechanistic analysis reveals different modes of interaction with the pyridoxal-5'-phosphate cofactor. *Journal of medicinal chemistry* **52**, 6032-6041, doi:10.1021/jm900775q (2009).

15 Dellafiora, L. *et al.* Expanding the chemical space of human serine racemase inhibitors. *Bioorganic & medicinal chemistry letters* **25**, 4297-4303, doi:10.1016/j.bmcl.2015.07.081 (2015).

16 Jiraskova-Vanickova, J. *et al.* Inhibition of human serine racemase, an emerging target for medicinal chemistry. *Curr Drug Targets* **12**, 1037-1055 (2011).

17 Vorlova, B. *et al.* Malonate-based inhibitors of mammalian serine racemase: kinetic characterization and structure-based computational study. *European journal of medicinal chemistry* **89**, 189-197, doi:10.1016/j.ejmech.2014.10.043 (2015).

18 Harty, M. *et al.* Inhibition of serine and proline racemases by substrate-product analogues. *Bioorganic & medicinal chemistry letters* **24**, 390-393, doi:10.1016/j.bmcl.2013.10.061 (2014).
19 Beato, C. *et al.* Cyclopropane derivatives as potential human serine racemase inhibitors: unveiling novel insights into a difficult target. *J Enzyme Inhib Med Chem*, 1-8, doi:10.3109/14756366.2015.1057720 (2015).
20 Dixon, S. M. *et al.* Slow-binding human serine racemase inhibitors from high-throughput screening of combinatorial libraries. *Journal of medicinal chemistry* **49**, 2388-2397, doi:10.1021/jm050701c (2006).
21 Mori, H. *et al.* In silico and pharmacological screenings identify novel serine racemase inhibitors. *Bioorganic & medicinal chemistry letters* **24**, 3732-3735, doi:10.1016/j.bmcl.2014.07.003 (2014).
22 Mori, H. *et al.* A novel serine racemase inhibitor suppresses neuronal over-activation in vivo. *Bioorg Med Chem* **25**, 3736-3745, doi:10.1016/j.bmc.2017.05.011 (2017).
23 Rani, K. *et al.* Accelerated identification of serine racemase inhibitor from Centella asiatica. *Scientific reports* **10**, 4640, doi:10.1038/s41598-020-61494-1 (2020).
24 Mustafa, A. K. *et al.* Glutamatergic regulation of serine racemase via reversal of PIP2 inhibition. *Proceedings of the National Academy of Sciences of the United States of America* **106**, 2921-2926, doi:10.1073/pnas.0813105106 (2009).
25 Kim, P. M. *et al.* Serine racemase: activation by glutamate neurotransmission via glutamate receptor interacting protein and mediation of neuronal migration. *Proceedings of the National Academy of Sciences of the United States of America* **102**, 2105-2110, doi:10.1073/pnas.0409723102 (2005).
26 Fujii, K. *et al.* Serine racemase binds to PICK1: potential relevance to schizophrenia. *Mol Psychiatry* **11**, 150-157, doi:10.1038/sj.mp.4001776 (2006).
27 Dumin, E. *et al.* Modulation of D-serine levels via ubiquitin-dependent proteasomal degradation of serine racemase. *J Biol Chem* **281**, 20291-20302, doi:10.1074/jbc.M601971200 (2006).
28 Mustafa, A. K. *et al.* Nitric oxide S-nitrosylates serine racemase, mediating feedback inhibition of D-serine formation. *Proceedings of the National Academy of Sciences of the United States of America* **104**, 2950-2955, doi:10.1073/pnas.0611620104 (2007).
29 Suzuki, M. *et al.* Glycolytic flux controls D-serine synthesis through glyceraldehyde-3-phosphate dehydrogenase in astrocytes. *Proc Natl Acad Sci U S A* **112**, E2217-2224, doi:10.1073/pnas.1416117112 (2015).
30 Bruno, S. *et al.* Human serine racemase is allosterically modulated by NADH and reduced nicotinamide derivatives. *Biochem J* **473**, 3505-3516, doi:10.1042/BCJ20160566 (2016).
31 Ma, T. M. *et al.* Serine racemase regulated by binding to stargazin and PSD-95: potential N-methyl-D-aspartate-α-amino-3-hydroxy-5-methyl-4-isoxazolepropionic acid (NMDA-AMPA) glutamate neurotransmission cross-talk. *J Biol Chem* **289**, 29631-29641, doi:10.1074/jbc.M114.571604 (2014).
32 Ma, T. M. *et al.* Pathogenic disruption of DISC1-serine racemase binding elicits schizophrenia-like behavior via D-serine depletion. *Mol Psychiatry* **18**, 557-567, doi:10.1038/mp.2012.97 (2013).
33 Graham, D. L., Beio, M. L., Nelson, D. L. & Berkowitz, D. B. Human Serine Racemase: Key Residues/Active Site Motifs and Their Relation to Enzyme Function. *Front Mol Biosci* **6**, 8, doi:10.3389/fmolb.2019.00008 (2019).
34 UniProt Consortium. UniProt: a worldwide hub of protein knowledge. *Nucleic acids research* **47**, doi:10.1093/nar/gky1049 (2019).
35 Foltyn, V. N., Zehl, M., Dikopoltsev, E., Jensen, O. N. & Wolosker, H. Phosphorylation of mouse serine racemase regulates D-serine synthesis. *FEBS Lett* **584**, 2937-2941, doi:10.1016/j.febslet.2010.05.022 (2010).

36 Balan, L. *et al.* Feedback inactivation of D-serine synthesis by NMDA receptor-elicited translocation of serine racemase to the membrane. *Proceedings of the National Academy of Sciences of the United States of America* **106**, 7589-7594, doi:10.1073/pnas.0809442106 (2009).
37 Balu, D. T., Takagi, S., Puhl, M. D., Benneyworth, M. A. & Coyle, J. T. D-serine and serine racemase are localized to neurons in the adult mouse and human forebrain. *Cell Mol Neurobiol* **34**, 419-435, doi:10.1007/s10571-014-0027-z (2014).
38 Coyle, J. T. & Balu, D. T. The Role of Serine Racemase in the Pathophysiology of Brain Disorders. *Adv Pharmacol* **82**, 35-56, doi:10.1016/bs.apha.2017.10.002 (2018).
39 Dememes, D., Mothet, J. P. & Nicolas, M. T. Cellular distribution of D-serine, serine racemase and D-amino acid oxidase in the rat vestibular sensory epithelia. *Neuroscience* **137**, 991-997, doi:10.1016/j.neuroscience.2005.09.020 (2006).
40 Jacobi, A. A., Halawani, S., Lynch, D. R. & Lin, H. Neuronal serine racemase associates with Disrupted-In-Schizophrenia-1 and DISC1 agglomerates: Implications for schizophrenia. *Neurosci Lett* **692**, 107-114, doi:10.1016/j.neulet.2018.10.055 (2019).
41 Mustafa, A. K., Kim, P. M. & Snyder, S. H. D-Serine as a putative glial neurotransmitter. *Neuron Glia Biol* **1**, 275-281, doi:10.1017/S1740925X05000141 (2004).
42 Vargas-Lopes, C. *et al.* Protein kinase C activity regulates D-serine availability in the brain. *J Neurochem* **116**, 281-290, doi:10.1111/j.1471-4159.2010.07102.x (2011).
43 Ma, T. *et al.* D-Serine Contributes to Seizure Development via ERK Signaling. *Front Neurosci* **13**, 254, doi:10.3389/fnins.2019.00254 (2019).
44 Ivanov, A. D. & Mothet, J. P. The plastic d-serine signaling pathway: Sliding from neurons to glia and vice-versa. *Neurosci Lett* **689**, 21-25, doi:10.1016/j.neulet.2018.05.039 (2019).
45 Dev, K. K. & Henley, J. M. The schizophrenic faces of PICK1. *Trends Pharmacol Sci* **27**, 574-579, doi:10.1016/j.tips.2006.09.007 (2006).
46 Boehning, D. & Snyder, S. H. Novel neural modulators. *Annu Rev Neurosci* **26**, 105-131, doi:10.1146/annurev.neuro.26.041002.131047 (2003).
47 Abazyan, S. *et al.* Mutant disrupted-in-schizophrenia 1 in astrocytes: focus on glutamate metabolism. *J Neurosci Res* **92**, 1659-1668, doi:10.1002/jnr.23459 (2014).
48 Xia, M., Zhu, S., Shevelkin, A., Ross, C. A. & Pletnikov, M. DISC1, astrocytes and neuronal maturation: a possible mechanistic link with implications for mental disorders. *J Neurochem* **138**, 518-524, doi:10.1111/jnc.13663 (2016).
49 Saitoh, Y. *et al.* d-Serine and d-Alanine Regulate Adaptive Foraging Behavior in Caenorhabditis elegans via the NMDA Receptor. *J Neurosci* **40**, 7531-7544, doi:10.1523/JNEUROSCI.2358-19.2020 (2020).
50 De Miranda, J., Santoro, A., Engelender, S. & Wolosker, H. Human serine racemase: moleular cloning, genomic organization and functional analysis. *Gene* **256**, 183-188 (2000).
51 Hoffman, H. E., Jiraskova, J., Ingr, M., Zvelebil, M. & Konvalinka, J. Recombinant human serine racemase: enzymologic characterization and comparison with its mouse ortholog. *Protein Expr Purif* **63**, 62-67, doi:10.1016/j.pep.2008.09.003 (2009).
52 Yoshimura, T. & Goto, M. D-amino acids in the brain: structure and function of pyridoxal phosphate-dependent amino acid racemases. *FEBS J* **275**, 3527-3537, doi:10.1111/j.1742-4658.2008.06516.x (2008).
53 Khersonsky, O. & Tawfik, D. S. Enzyme promiscuity: a mechanistic and evolutionary perspective. *Annu Rev Biochem* **79**, 471-505, doi:10.1146/annurev-biochem-030409-143718 (2010).
54 Dajnowicz, S. *et al.* Direct visualization of critical hydrogen atoms in a pyridoxal 5'-phosphate enzyme. *Nat Commun* **8**, 955, doi:10.1038/s41467-017-01060-y (2017).
55 Bax, B., Chung, C. W. & Edge, C. Getting the chemistry right: protonation, tautomers and the importance of H atoms in biological chemistry. *Acta Crystallogr D Struct Biol* **73**, 131-140, doi:10.1107/S2059798316020283 (2017).

56 Ito, T. *et al.* Metal ion dependency of serine racemase from Dictyostelium discoideum. *Amino Acids* **43**, 1567-1576, doi:10.1007/s00726-012-1232-z (2012).

57 Marchetti, M. *et al.* ATP binding to human serine racemase is cooperative and modulated by glycine. *FEBS J* **280**, 5853-5863, doi:10.1111/febs.12510 (2013).

58 Canosa, A. V. *et al.* Glutamine 89 is a key residue in the allosteric modulation of human serine racemase activity by ATP. *Scientific reports* **8**, 9016, doi:10.1038/s41598-018-27227-1 (2018).

59 Wolosker, H. Serine racemase and the serine shuttle between neurons and astrocytes. *Biochim Biophys Acta* **1814**, 1558-1566, doi:10.1016/j.bbapap.2011.01.001 (2011).

60 Perez, E. J. *et al.* Enhanced astrocytic d-serine underlies synaptic damage after traumatic brain injury. *J Clin Invest* **127**, 3114-3125, doi:10.1172/JCI92300 (2017).

61 Balu, D. T. *et al.* Neurotoxic astrocytes express the d-serine synthesizing enzyme, serine racemase, in Alzheimer's disease. *Neurobiol Dis* **130**, 104511, doi:10.1016/j.nbd.2019.104511 (2019).

62 Foltyn, V. N. *et al.* Serine racemase modulates intracellular D-serine levels through an alpha,beta-elimination activity. *J Biol Chem* **280**, 1754-1763, doi:10.1074/jbc.M405726200 (2005).
