# Supplementary material for: Tyrosine 121 moves revealing a ligandable pocket that couples catalysis to ATP-binding in serine racemase
Source: Commun Biol. 2022 Apr 11;5:346. doi: 10.1038/s42003-022-03264-5 (PMC9001717; doi:10.1038/s42003-022-03264-5)
Supplement: Supplementary file 3 — Description of Additional Supplementary Files [file 42003_2022_3264_MOESM3_ESM.pdf]

Description of Additional Supplementary Files

File name: Supplementary Movie S1
Description: Comparison of dimer in 'open' and 'closed' structures.

File name: Supplementary Movie S2
Description: View of movement of tyrosine 121.

File name: Supplementary Movie S3
Description: View of movement of catalytic serine 84.
