# Supplementary material for: Tyrosine 121 moves revealing a ligandable pocket that couples catalysis to ATP-binding in serine racemase
Source: Commun Biol. 2022 Apr 11;5:346. doi: 10.1038/s42003-022-03264-5 (PMC9001717; doi:10.1038/s42003-022-03264-5)
Supplement: Supplementary file 7 — Reporting summary [file 42003_2022_3264_MOESM7_ESM.pdf]

nature portfolio

Corresponding author(s): Ben Bax and John Atack

Last updated by author(s): Feb 1, 2022

Reporting Summary

Nature Portfolio wishes to improve the reproducibility of the work that we publish. This form provides structure for consistency and transparency in reporting. For further information on Nature Portfolio policies, see our Editorial Policies and the Editorial Policy Checklist.

Please do not complete any field with "not applicable" or n/a. Refer to the help text for what text to use if an item is not relevant to your study.
For final submission: please carefully check your responses for accuracy; you will not be able to make changes later.

Statistics

For all statistical analyses, confirm that the following items are present in the figure legend, table legend, main text, or Methods section.

n/a	Confirmed	
☐	☒	The exact sample size (n) for each experimental group/condition, given as a discrete number and unit of measurement
☐	☒	A statement on whether measurements were taken from distinct samples or whether the same sample was measured repeatedly
☐	☒	The statistical test(s) used AND whether they are one- or two-sided<br>*Only common tests should be described solely by name; describe more complex techniques in the Methods section.*
☒	☐	A description of all covariates tested
☒	☐	A description of any assumptions or corrections, such as tests of normality and adjustment for multiple comparisons
☐	☒	A full description of the statistical parameters including central tendency (e.g. means) or other basic estimates (e.g. regression coefficient) AND variation (e.g. standard deviation) or associated estimates of uncertainty (e.g. confidence intervals)
☒	☐	For null hypothesis testing, the test statistic (e.g. F, t, r) with confidence intervals, effect sizes, degrees of freedom and P value noted<br>*Give P values as exact values whenever suitable.*
☒	☐	For Bayesian analysis, information on the choice of priors and Markov chain Monte Carlo settings
☒	☐	For hierarchical and complex designs, identification of the appropriate level for tests and full reporting of outcomes
☒	☐	Estimates of effect sizes (e.g. Cohen's d, Pearson's r), indicating how they were calculated

Our web collection on statistics for biologists contains articles on many of the points above.

Software and code

Policy information about availability of computer code

Data collection	The diffraction images were processed using the Xia2 software pipeline 61: DIALS 62 for data processing, POINTLESS and AIMLESS 63 for symmetry determination and scaling, and CTRUNCATE 64 for calculation of amplitudes and intensities.
Data analysis	Processing and refinement were carried out using the CCP4 65 and phenix 66 suite of programmes. The structure of the hSR complex with malonate and ATP (pdb code: 6ZSP) was solved by molecular replacement in MOLREP 67 using the crystal structure from the previously determined malonate-bound structure (pdb code: 3L6B) with iterative refinement in REFMAC5 68 and manual model building in Coot 69.

For manuscripts utilizing custom algorithms or software that are central to the research but not yet described in published literature, software must be made available to editors and reviewers. We strongly encourage code deposition in a community repository (e.g. GitHub). See the Nature Portfolio guidelines for submitting code & software for further information.

Data

Policy information about availability of data

All manuscripts must include a data availability statement. This statement should provide the following information, where applicable:

- Accession codes, unique identifiers, or web links for publicly available datasets
- A description of any restrictions on data availability
- For clinical datasets or third party data, please ensure that the statement adheres to our policy

The coordinates and structure factors have been deposited in the Protein Data Bank. Details of the structures with accession codes (6ZSP for Mal/ATP) and (6ZUJ XChem apo, 7NBC, 7NBD, 7NBF, 7NBG, 7NBH, XChem fragment soaks) are listed in Supplementary Table 1.

Field-specific reporting

Please select the one below that is the best fit for your research. If you are not sure, read the appropriate sections before making your selection.

☒ Life sciences ☐ Behavioural & social sciences ☐ Ecological, evolutionary & environmental sciences

Life sciences study design

All studies must disclose on these points even when the disclosure is negative.

Sample size	Sample sizes determined using standard crystallographic data processing software - as show in supplementary table 1 and explained in methods
Data exclusions	No exclusions. Intensities were converted to structure factor amplitudes using CTRUNCATE as explained in methods.
Replication	We did not attempt to replicate crystal structures with fragments.
Randomization	We did not do this. We used standard crystallographic methods - including R-free.
Blinding	We did not do this. We used standard crystallographic methods - including R-free

Reporting for specific materials, systems and methods

We require information from authors about some types of materials, experimental systems and methods used in many studies. Here, indicate whether each material, system or method listed is relevant to your study. If you are not sure if a list item applies to your research, read the appropriate section before selecting a response.

Materials & experimental systems

n/a	Involved in the study
☒	☐ Antibodies
☒	☐ Eukaryotic cell lines
☒	☐ Palaeontology and archaeology
☒	☐ Animals and other organisms
☒	☐ Human research participants
☒	☐ Clinical data
☒	☐ Dual use research of concern

Methods

n/a	Involved in the study
☒	☐ ChIP-seq
☒	☐ Flow cytometry
☒	☐ MRI-based neuroimaging

This checklist template is licensed under a Creative Commons Attribution 4.0 International License, which permits use, sharing, adaptation, distribution and reproduction in any medium or format, as long as you give appropriate credit to the original author(s) and the source, provide a link to the Creative Commons license, and indicate if changes were made. The images or other third party material in this article are included in the article's Creative Commons license, unless indicated otherwise in a credit line to the material. If material is not included in the article's Creative Commons license and your intended use is not permitted by statutory regulation or exceeds the permitted use, you will need to obtain permission directly from the copyright holder. To view a copy of this license, visit http://creativecommons.org/licenses/by/4.0/
